# An atlas of dynamic chromatin landscapes in mouse fetal development

**DOI:** 10.1038/s41586-020-2093-3

**Published:** 2020-07-29

**Authors:** David U. Gorkin, Iros Barozzi, Yuan Zhao, Yanxiao Zhang, Hui Huang, Ah Young Lee, Bin Li, Joshua Chiou, Andre Wildberg, Bo Ding, Bo Zhang, Mengchi Wang, J. Seth Strattan, Jean M. Davidson, Yunjiang Qiu, Veena Afzal, Jennifer A. Akiyama, Ingrid Plajzer-Frick, Catherine S. Novak, Momoe Kato, Tyler H. Garvin, Quan T. Pham, Anne N. Harrington, Brandon J. Mannion, Elizabeth A. Lee, Yoko Fukuda-Yuzawa, Yupeng He, Sebastian Preissl, Sora Chee, Jee Yun Han, Brian A. Williams, Diane Trout, Henry Amrhein, Hongbo Yang, J. Michael Cherry, Wei Wang, Kyle Gaulton, Joseph R. Ecker, Yin Shen, Diane E. Dickel, Axel Visel, Len A. Pennacchio, Bing Ren

**Affiliations:** 1 Ludwig Institute for Cancer Research, La Jolla, CA USA; 2 Center for Epigenomics, University of California, San Diego School of Medicine, La Jolla, CA USA; 3 Environmental Genomics and Systems Biology Division, Lawrence Berkeley National Laboratory, Berkeley, CA USA; 4 Department of Surgery and Cancer, Imperial College London, London, UK; 5 Bioinformatics and Systems Biology Graduate Program, University of California, San Diego, La Jolla, CA USA; 6 Biomedical Sciences Graduate Program, University of California, San Diego School of Medicine, La Jolla, CA USA; 7 Department of Pediatrics, University of California, San Diego School of Medicine, La Jolla, CA USA; 8 Department of Cellular and Molecular Medicine, University of California, San Diego School of Medicine, La Jolla, CA USA; 9 Department of Biochemistry and Molecular Biology, Penn State School of Medicine, Hershey, PA USA; 10 Stanford University School of Medicine, Department of Genetics, Stanford, CA USA; 11 Genomic Analysis Laboratory, Salk Institute for Biological Studies, La Jolla, CA USA; 12 Division of Biology and Biological Engineering, California Institute of Technology, Pasadena, CA USA; 13 Howard Hughes Medical Institute, Salk Institute for Biological Studies, La Jolla, CA USA; 14 Institute for Human Genetics and University of California, San Francisco, San Francisco, CA USA; 15 Department of Neurology, University of California, San Francisco, San Francisco, CA USA; 16 US Department of Energy Joint Genome Institute, Berkeley, CA USA; 17 School of Natural Sciences, University of California, Merced, Merced, CA USA; 18 Comparative Biochemistry Program, University of California, Berkeley, Berkeley, CA USA; 19 Institute of Genomic Medicine, University of California, San Diego School of Medicine, La Jolla, CA USA; 20 Moores Cancer Center, University of California, San Diego School of Medicine, La Jolla, CA USA

**Keywords:** Epigenomics, Differentiation

## Abstract

The Encyclopedia of DNA Elements (ENCODE) project has established a genomic resource for mammalian development, profiling a diverse panel of mouse tissues at 8 developmental stages from 10.5 days after conception until birth, including transcriptomes, methylomes and chromatin states. Here we systematically examined the state and accessibility of chromatin in the developing mouse fetus. In total we performed 1,128 chromatin immunoprecipitation with sequencing (ChIP–seq) assays for histone modifications and 132 assay for transposase-accessible chromatin using sequencing (ATAC–seq) assays for chromatin accessibility across 72 distinct tissue-stages. We used integrative analysis to develop a unified set of chromatin state annotations, infer the identities of dynamic enhancers and key transcriptional regulators, and characterize the relationship between chromatin state and accessibility during developmental gene regulation. We also leveraged these data to link enhancers to putative target genes and demonstrate tissue-specific enrichments of sequence variants associated with disease in humans. The mouse ENCODE data sets provide a compendium of resources for biomedical researchers and achieve, to our knowledge, the most comprehensive view of chromatin dynamics during mammalian fetal development to date.

## Abstract (web summary)

Analysis of chromatin state and accessibility in mouse tissues from twelve sites and eight developmental stages provides a comprehensive view of chromatin dynamics.

## Main

Developmental gene regulation relies on a complex interplay between genetic and epigenetic factors. Whereas genetic information encoded in the DNA sequence provides the instructions for an embryo to develop, epigenetic information is required for each cell in an embryo to obtain its specialized function from this single set of instructions. Chromatin encodes epigenetic information in the form of post-translational histone modifications and accessibility to DNA binding factors^1,2^. Developmental programs of gene expression are orchestrated, at least in part, by *cis*-regulatory sequences that direct the expression of genes in response to specific developmental and environmental cues^3,4^. Active regulatory sequences show characteristic patterns of histone modification and accessible chromatin that make them amenable to the binding of transcription factors (TFs), which can in turn recruit co-factors and stimulate transcription. These epigenomic properties have proven valuable for genome annotation, because histone modifications and accessibility at a given genome region can reflect the activity of the underlying sequence^5,6^.

In previous phases of the ENCODE project, epigenomic and transcriptomic data sets were generated from mouse tissues at a single prenatal time point (embryonic day (E)14.5) and two postnatal time points (8 and 24 weeks after birth)^5^. In the most recent phase of ENCODE, we made a coordinated effort to create resources for the study of mammalian fetal development by generating epigenomic and transcriptomic data sets from 7 additional stages of fetal development covering a window from E10.5 until birth at approximately one-day intervals. At each stage, we collected a diverse panel of 8–12 tissues to make a total of 72 tissue-stages, with 2 biological replicates per tissue-stage, and each replicate containing tissue pooled from multiple embryos. This common tissue resource was used as input for RNA sequencing (RNA-seq)^98^, whole-genome bisulfite sequencing^7^, ATAC–seq, and ChIP–seq for eight histone modifications (ATAC–seq and ChIP–seq described here). Data from this and all phases of ENCODE are publicly available through the ENCODE portal (https://www.encodeproject.org/).

To map chromatin states during mouse fetal development, we performed ChIP–seq for a set of eight histone modifications that can distinguish between functional elements and activity levels. To assay chromatin accessibility, we used a version of ATAC–seq^8^ optimized for use on frozen tissues (Methods). Chromatin accessibility can also be mapped by DNase I hypersensitive sites sequencing (DNase-seq), which has been integral to the identification of millions of candidate regulatory sequences in mammalian genomes^9,10^, but we chose ATAC–seq here because it offers a more streamlined workflow. The resulting maps of chromatin accessibility, together with those of histone modifications, provide deep insight into the genomic regions and processes that drive mouse fetal development.•

- We systematically map chromatin state and accessibility across 72 distinct tissue-stages of mouse development, and carry out integrative analyses incorporating additional epigenomic and transcriptomic data sets from the same tissue-stages.
- We derive a chromatin state model from combinatorial patterns of histone modifications, encompassing 15 distinct states grouped in 4 broad functional classes: promoter, enhancer, transcriptional, and heterochromatin states.
- We characterize the spatial and temporal dynamics of chromatin states, finding that approximately 1–4% of the genome differs in chromatin state between tissues at the same stage, and 0.03–3% differs between adjacent stages of the same tissue; enhancer chromatin states show the largest differences in both cases.
- We show that Polycomb-mediated repression is pervasive during fetal development at genes that encode transcriptional regulators and enriched at those with human orthologues linked to Mendelian diseases.
- We identify more than 500,000 developmental regions of transposase-accessible chromatin marked by accessible chromatin during mouse fetal development, including approximately 140,000 with dynamic temporal activity in at least one tissue.
- We show that human orthologues of mouse fetal accessible chromatin regions are enriched for human disease-associated sequence variation, with apparent tissue-restricted patterns of enrichment.
- We show that temporal changes in chromatin accessibility often coincide with changes in enhancer chromatin states, and tend to precede changes in nearby H3K27ac levels.
- We predict 21,142 enhancer–promoter interactions by measuring the correlation between enhancer-associated chromatin signals and gene expression across tissues-stages.
- We show that candidate enhancers with stronger enrichment for marks of regulatory activity such as H3K27ac show a higher validation rate in reporter assays in vivo.

## Profiling chromatin states in vivo

**Fig. 1:**
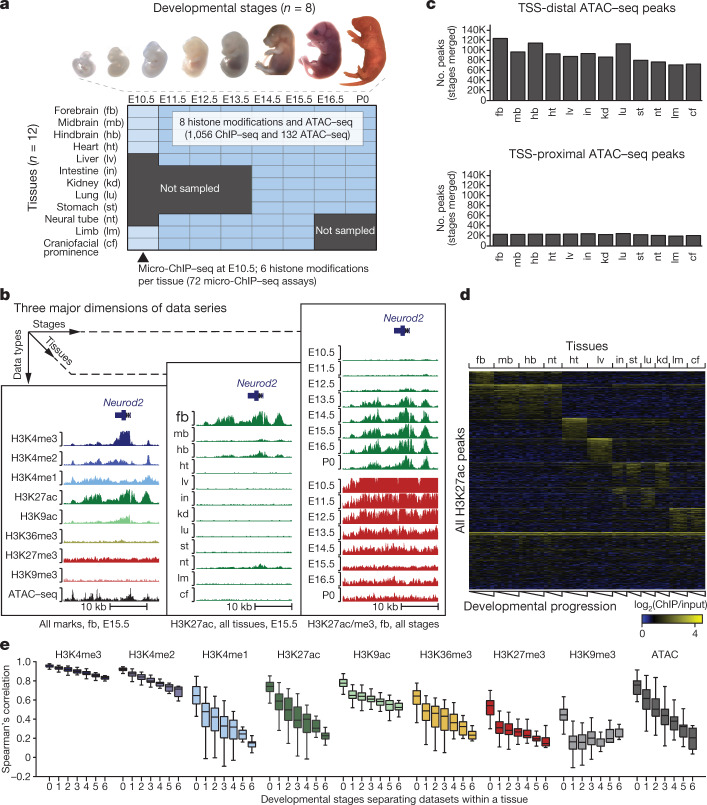
Profiling histone modifications during mouse fetal development. **a**, Experimental design. **b**, Three major axes of the data series: data types, tissues, and developmental stages (chr11: 98,318,134–98,336,928; mm10). Horizontal scale 0–30 for narrow marks (H3K4me3, H3K4me2, H3K27ac, H3K9ac), 0–10 for broad marks (H3K27me3, H3K4me1, H3K9me3, H3K36me3) and ATAC–seq. **c**, Number of TSS-distal (top, >1 kb) and TSS-proximal (bottom) ATAC–seq peaks for each tissue. **d**, *k*-means clustering of H3K27ac peaks (*n* = 333,097) across tissue-stages (*k* = 8). Cluster sizes, top to bottom: 20,497, 50,790, 31,043, 36,849, 38,670, 31,168, 36,822 and 87,258. **e**, Spearman’s correlations of peak strength between replicates from the same stage (that is, developmental stages separating data sets is 0), or from different stages separated by one to six intervening stages, as indicated. Number of points per comparison: 0 stages, 66; 1 stage, 108; 2 stages, 84; 3 stages, 60; 4 stages, 36; 5 stages, 20; 6 stages, 10. For all boxplots in this paper: horizontal line, median; box, interquartile range (IQR); whiskers, most extreme value within ±1.5 × IQR.

**Extended Data Fig. 1:**
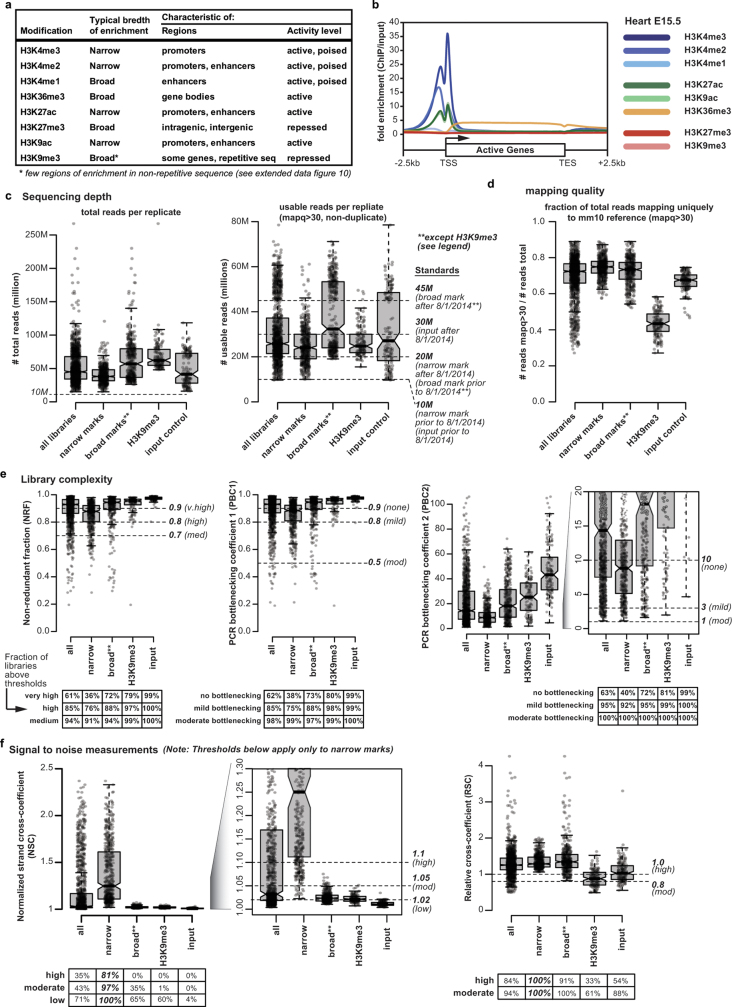
ChIP–seq data summary. **a**, Summary of characteristic enrichment patterns for histone modifications surveyed here. Modifications are generally categorized as narrow or broad depending on the typical breadth of enrichment. H3K9me3 is further distinguished from other broad marks because it shows very few regions of enrichment in non-repetitive sequence in primary tissues and cells^26^. **b**, Metagene plot illustrating the typical patterns of histone modification enrichment at active genes (here defined as RPKM >10 in all tissue-stages surveyed). ChIP–seq data plotted are from embryonic heart at E15.5. **c**, Sequencing depth plotted for every library reported (*n* = 1,272 total, 552 narrow, 432 broad, 144 H3K9me3, 144 input). ENCODE ‘usable’ read depth standards (mapping quality scores (mapq) >30, and after PCR duplicate removal) are indicated to the right. Read depth standards changed part way through our study (increasing from 10M to 20M for narrow marks, 20M to 45M for broad marks, and 10M to 30M for input). All narrow mark libraries exceed the 10M minimal depth. Broad mark libraries exceed the 20M minimal depth with only four exceptions, all of which exceed 19M. Input libraries exceed the 10M minimal depth with only one exception, which exceeds 9.7M. The read depth standard for H3K9me3 is >45M mapped reads of any mapq (because H3k9me3 is enriched in repetitive sequence, Extended Data Fig. 10); all H3K9me3 libraries exceed this threshold. Box plots: horizontal line, median; box, IQR; whiskers, most extreme value within ±1.5 × IQR. **d**, Mapping quality plotted for every library, measured as the fraction of reads with mapq >30. Reads with lower mapq scores (that is, non-uniquely mapping reads) were eliminated from downstream analysis. **e**, Three metrics of library complexity are plotted (NRF, PBC1, PBC2). See ENCODE data standards^90^ for detailed descriptions and formulas. Tables below each plot show the percentage of libraries that exceed the thresholds indicated. **f**, Two measures of signal-to-noise ratio are plotted (NSC, RSC). Again, detailed descriptions are available in the ENCODE data standards descriptions. These metrics are not well calibrated for broad marks or input and thresholds apply only to narrow marks.

**Extended Data Fig. 2:**
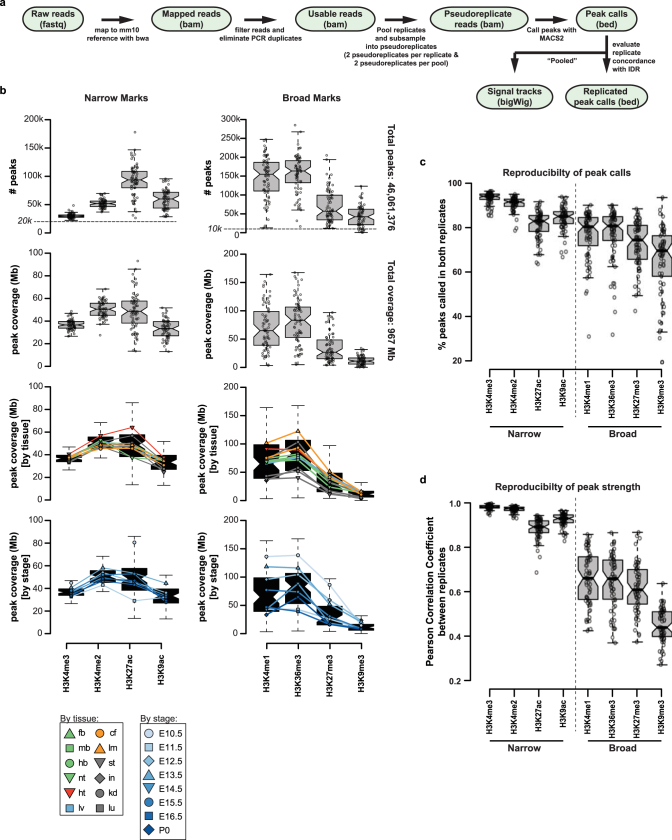
ChIP–seq peak calling. **a**, Schematic of ChIP–seq peak calling pipeline. More information can be found here: https://www.encodeproject.org/pipelines/. **b**, Four peak summary statistics plotted for every tissue-stage. From top to bottom: 1) number of peaks called (passing IDR threshold); 2) total coverage of those peaks; 3) peak coverage as in (2), but separated according to tissue; 4) peak coverage as in (2), but separated by stage. E10.5 ChIP–seq experiments were performed with a modified protocol, and in some cases a different, more sensitive antibody was used (H3K27ac, H3K4me1). We suspect that is why E10.5 sometimes appears as an outlier in terms of coverage. *n* = 72 for all marks, expect for H3K4me2 and H3K9ac where *n* = 66. **c**, Peak reproducibility as measured by the percentage of peaks called from the pooled data that were called independently in both individual replicates. **d**, Peak reproducibility as measured by correlation of peak strengths (average fold enrichment over input) between biological replicates.

**Extended Data Fig. 3:**
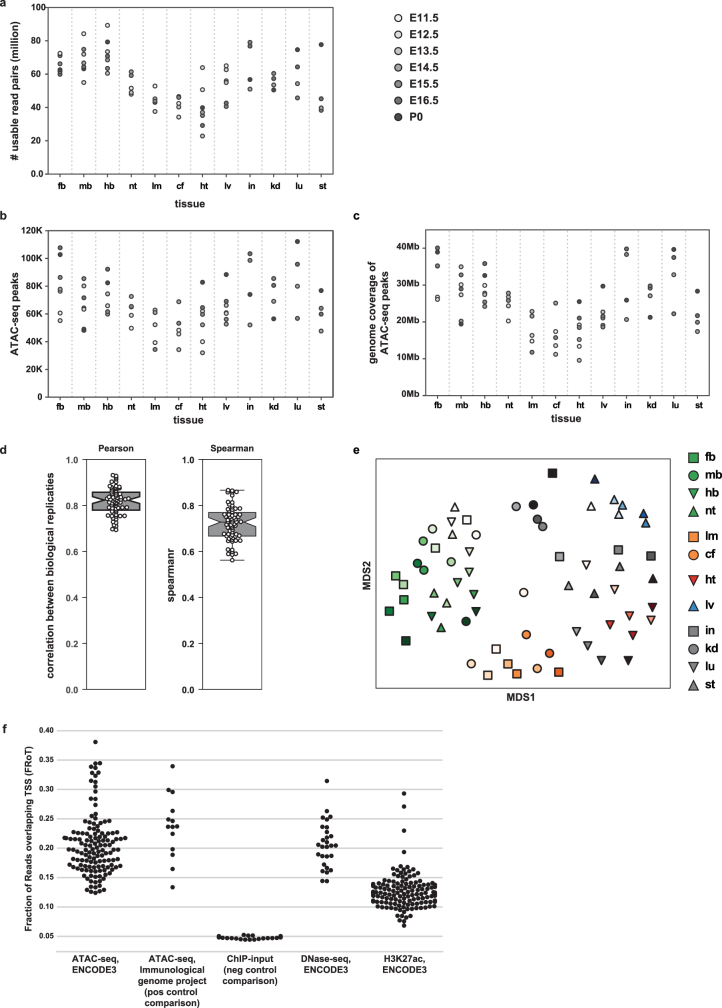
ATAC–seq data summary. **a**, The number of usable read pairs per tissue-stage, after filtering for mapping quality and PCR duplicates. **b**, The number of replicated ATAC–seq peaks called per tissue-stage. **c**, Genome coverage of replicated ATAC–seq peaks at each tissue-stage. **d**, Correlation of ATAC–seq signal at replicated peaks between biological replicates (*n* = 66 tissue-stages), as measured by Pearson’s correlation coefficient (left) or Spearman’s correlation coefficient (right). **e**, Multidimensional scaling (MDS) plot showing that the ATAC–seq signals at d-TACs tend to separate the samples first by tissue (indicated by coloured shapes) and then by stage (shade of colour within shapes). **f**, Fraction of usable reads overlapping TSS (measure of signal-to-noise ratio) for the ATAC–seq data and other reference data. H3K27ac ChIP–seq data and input from our ENCODE3 mouse tissues are shown to provide additional context for interpreting these numbers.

Despite the importance of chromatin states and accessibility in determining the functional output of the genome, a comprehensive survey of chromatin dynamics during mammalian fetal development has been lacking aside from very early stages of embryogenesis^11,12^. To address this gap, we collected mouse tissues at closely spaced intervals from E11.5 until birth. At each stage, we dissected a diverse panel of tissues from multiple litters of embryos and performed two replicates of ATAC–seq and ChIP–seq for each of eight histone modifications chosen to distinguish between different types of functional elements (for example, promoters, enhancers and gene bodies), and activity levels (for example, active, poised and repressed)^13,14^ (Fig. 1a, b, Extended Data Fig. 1a, b). We also profiled 6 tissues at E10.5, using a micro-ChIP–seq procedure designed for smaller cell numbers and restricting our scope to 6 histone modifications^15^. All ChIP–seq and ATAC–seq data sets were processed with a uniform pipeline and subjected to quality standards (Methods; Fig. 1c, Extended Data Figs. 1c–f, 2, 3). Whole-genome bisulfite sequencing and RNA-seq from other groups are reported in companion manuscripts^7,98^ and used in select analyses below.

**Extended Data Fig. 4:**
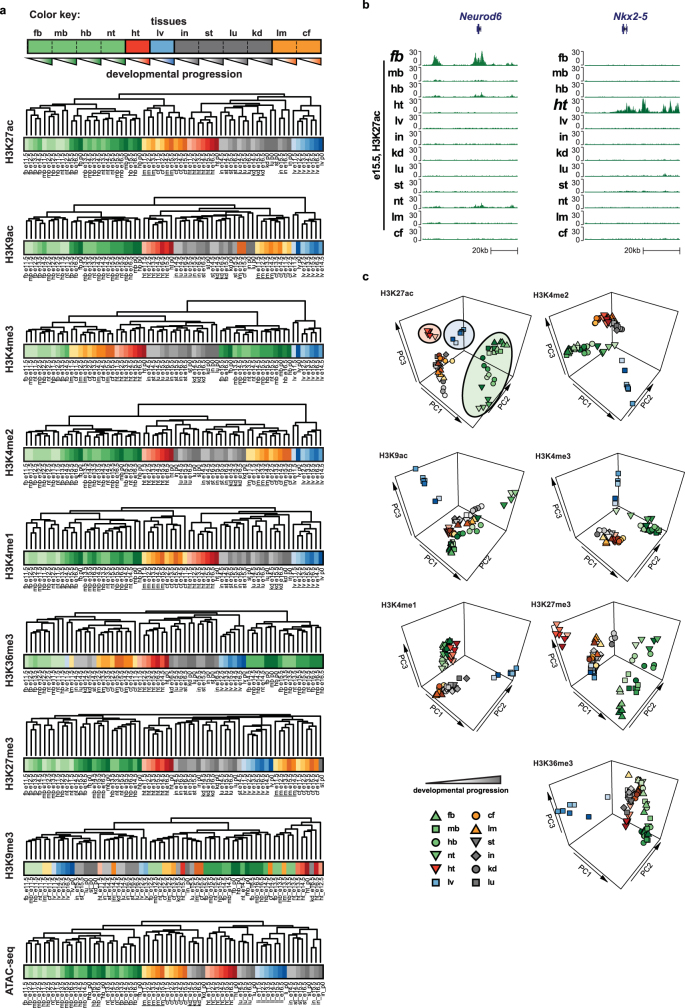
Chromatin landscapes across tissues. **a**, Dendrograms from hierarchical clustering based on signal for each histone modification and ATAC–seq, as indicated. Note the consistent relationships between tissues of similar developmental origin. **b**, Genome browser view of *NeuroD6* (chr6: 55,637,617–55,708,251; mm10) and *Nkx2–5* (chr17: 26,818,483–26,870,007; mm10), markers of neuronal and cardiomyocyte differentiation, respectively. **c**, Principal component analysis of all tissue-stages based on ChIP–seq data for individual histone modifications, as indicated.

**Extended Data Fig. 5:**
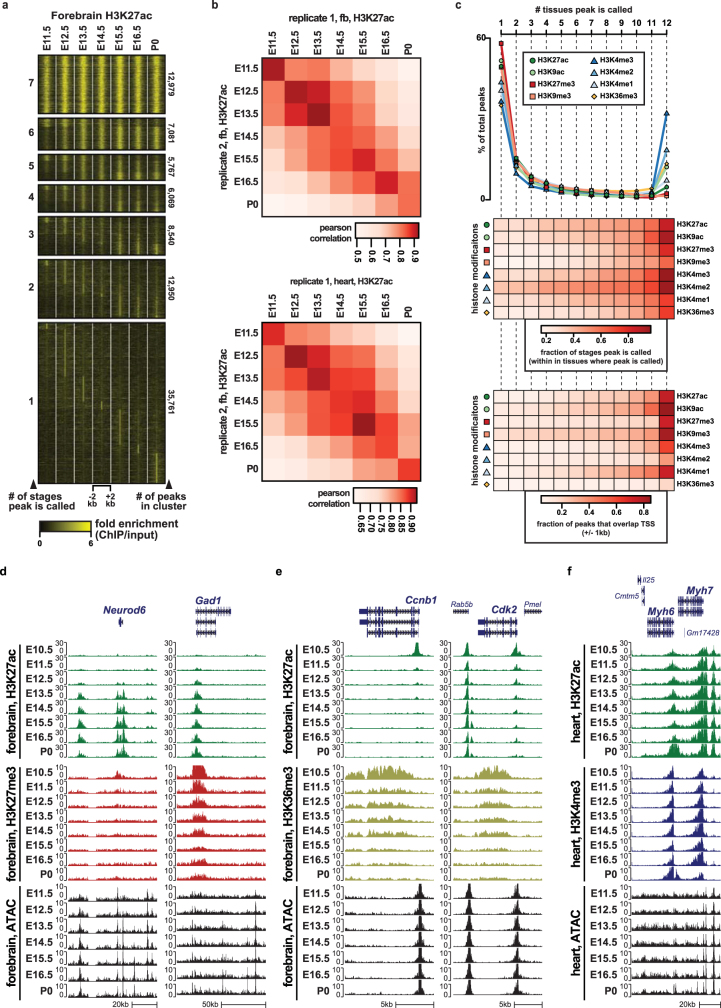
Chromatin landscapes across stages. **a**, Heatmap showing the H3K27ac ChIP–seq signal at H3K27ac ChIP–seq peaks in forebrain. Peaks are clustered according to how many stages within forebrain they were present at (*y*-axis, left). The number of peaks in each cluster is indicated to the right. **b**, Pearson’s correlation coefficients between H3K27ac signal in peaks at stages E11.5–P0 in forebrain (top) or heart (bottom). **c**, The *x*-axis at the top indicates the number of tissues in which a given peak is present (1–12). The top line plot shows tissue specificity as the percentage of total peaks for a given mark that were called in a given number of tissues. The middle heatmap shows stage specificity as the average fraction of stages within a tissue at which a peak is present. Peaks that are more restricted to specific tissues are also more restricted to specific stages within those tissues. The bottom heatmap shows the locations of peaks relative to TSSs by plotting the fraction of peaks that overlap an annotated GENCODE TSS. Peaks that are more consistent across tissues and across stages also tend to overlap a TSS. **d**, Genome browser view of *Gad1* (chr2: 70,547,104–70,615,401; mm10) and *NeuroD6* (chr6: 55,637,617–55,708,251; mm10), neuronal markers, showing the gain of active chromatin signatures during forebrain development. **e**, Genome browser views of *Ccnb1* (chr13: 100,776,802–100,788,423; mm10) and *Cdk2* (chr10: 128,693,493–128,709,497; mm10), key cell cycle regulators, showing the loss of active chromatin signatures during forebrain development. **f**, Genome browser view of the *Myh6*/*Myh7* locus (chr14: 54,927,121–55,010,762; mm10), showing a shift in activity from *Myh7* to *Myh6* that is known to occur in cardiomyocytes just before birth^91^.

We observed several notable high-level features of the data series. As expected, the landscape of histone modifications and chromatin accessibility varies between tissues, particularly for marks of activity such as H3K27ac (acetylation at the 27th lysine residue of histone H3) (Fig. 1d, Extended Data Fig. 4). Within each tissue, chromatin landscapes change progressively across stages (Fig. 1e, Extended Data Fig. 5a–c). These developmental dynamics are likely to reflect at least two underlying biological processes: changes in the epigenetic landscape of individual cells within a tissue as they undergo differentiation, and shifts in the relative abundance of different cell types that compose a tissue. Although in most cases we cannot separate the relative contributions of these two factors, many of the changes we observe reflect known hallmarks of cellular differentiation. For example, in the developing forebrain, neuronal markers acquire active chromatin signatures during development, whereas genes that encode cell cycle factors show the opposite trend (Fig. 1b, Extended Data Fig. 5d–f).

## The developmental chromatin landscape

**Fig. 2:**
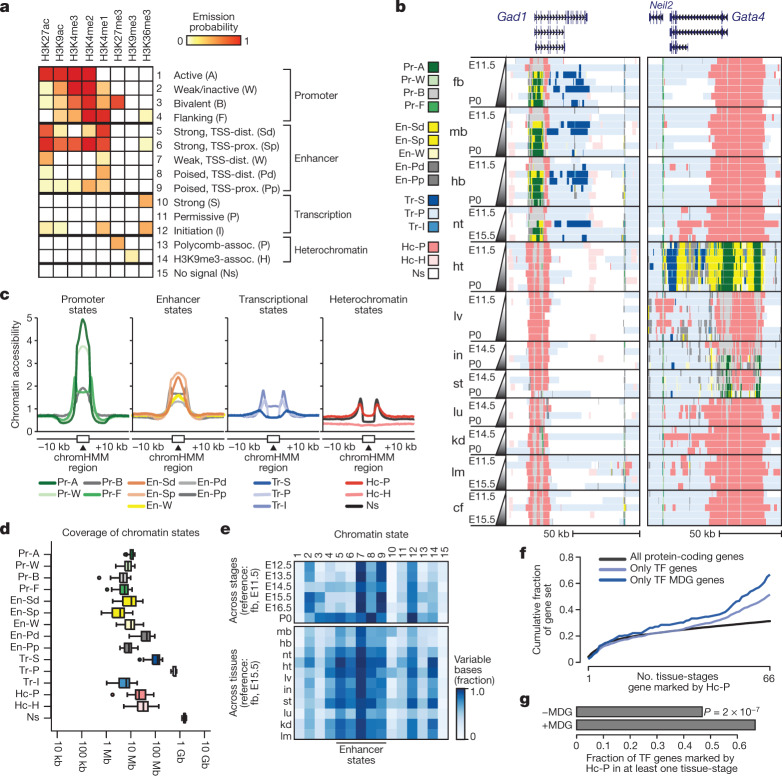
A 15-state model characterizes the mouse developmental chromatin landscape. **a**, Emission probabilities for histone modifications in 15 ChromHMM states, with descriptive title of each state. **b**, Chromatin state landscapes at *Gad1* (chr2: 70,541,017–70,641,016; mm10) and *Gata4* (chr14: 63,181,234–63,288,624; mm10). Pr, promoter; En, enhancer; Tr, transcription; Hc, heterochromatin. **c**, Average chromatin accessibility at different chromatin states in E15.5 forebrain. **d**, Genome coverage of chromatin states in each tissue-stage (*n* = 66). **e**, Fraction of bases for each state that vary in forebrain between E11.5 and other stages (top), or between E15.5 forebrain and other tissues at E15.5 (bottom). **f**, Fraction of indicated gene sets that show evidence of PcG repression: for all protein-coding genes (0.313, black line); TF protein-coding genes (0.515, light blue line); and MDG TF protein-coding genes (0.667, dark blue line). Cumulative fractions plotted by the number of tissue-stages at which a gene shows PcG repression (from one to 66, *x*-axis). **g**, MDG TFs are more likely to show evidence of PcG repression (MDG+, 150/225; MDG−, 349/744). *χ*^2^ test of independence between PcG repression and MDG involvement.

**Extended Data Fig. 6:**
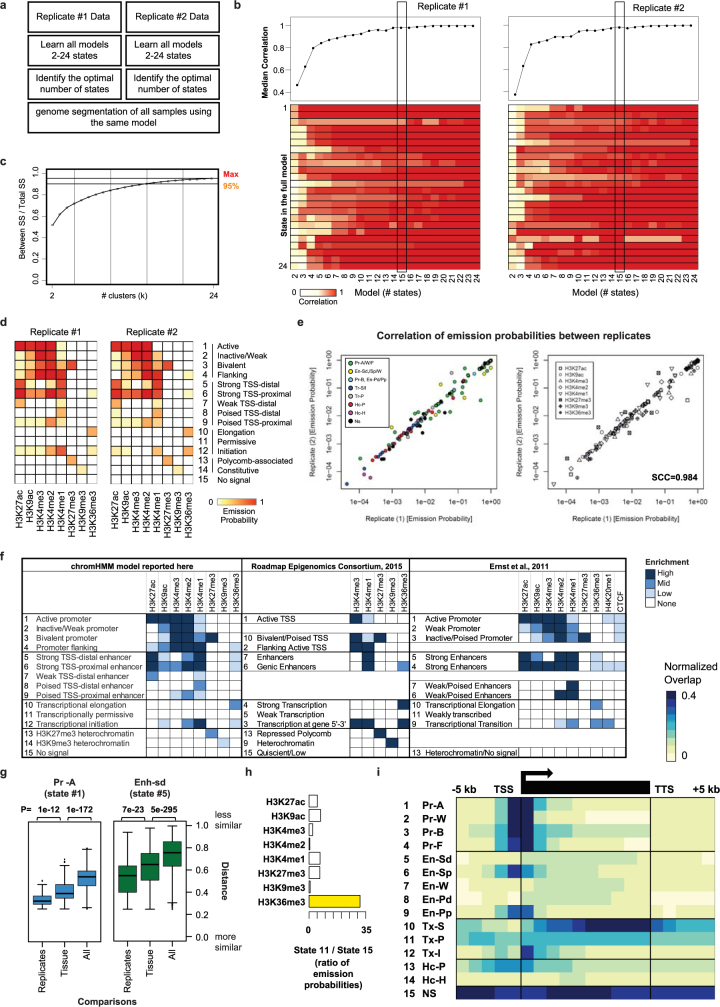
Fifteen-state ChromHMM model. **a**, Schematic of the ChromHMM strategy applied in this study. **b**, Heatmaps showing the maximum Pearson’s correlation of each state in the full model (*y*-axis) with its best matching state in each simpler model (*x*-axis). The median correlation of all 24 states is shown in the plots on top of the heatmaps. **c**, Classification of the *k*-means clustering of the emission probabilities from all the models. The optimal number of states was defined by the smallest value of *k* that showed a ratio equal to or higher than 95% (orange line) of the maximum clusters’ separation (red line). SS, sum of squares. **d**, The emission probabilities for each chromatin mark in each state, as defined by ChromHMM, for both replicates. **e**, Spearman’s correlation of emission probabilities from ChromHMM models derived from two biological replicates, colour-coded by state (left) or by modification (right). **f**, Comparison of the ChromHMM model reported here with previously published ChromHMM models. Horizontal white bars indicate chromatin states identified in our study that did not have a clear counterpart in those studies. **g**, Similarity between replicates from the same tissue-stage (*n* = 66), from the same tissue any stage (*n* = 702), or from any tissue any stage (*n* = 8,646). Similarity measured as pairwise binary distance. Two-sided Mann–Whitney test. **h**, Enrichment of each mark in state 11 (permissive) relative to state 15 (no signal, genomic background). The ChromHMM emission probability for H3K36me3 in state 11 is >30-fold higher than genomic background. **i**, Enrichment of chromatin states relative to annotated genes. Gene annotations were not considered during model training or genome segmentation.

**Extended Data Fig. 7:**
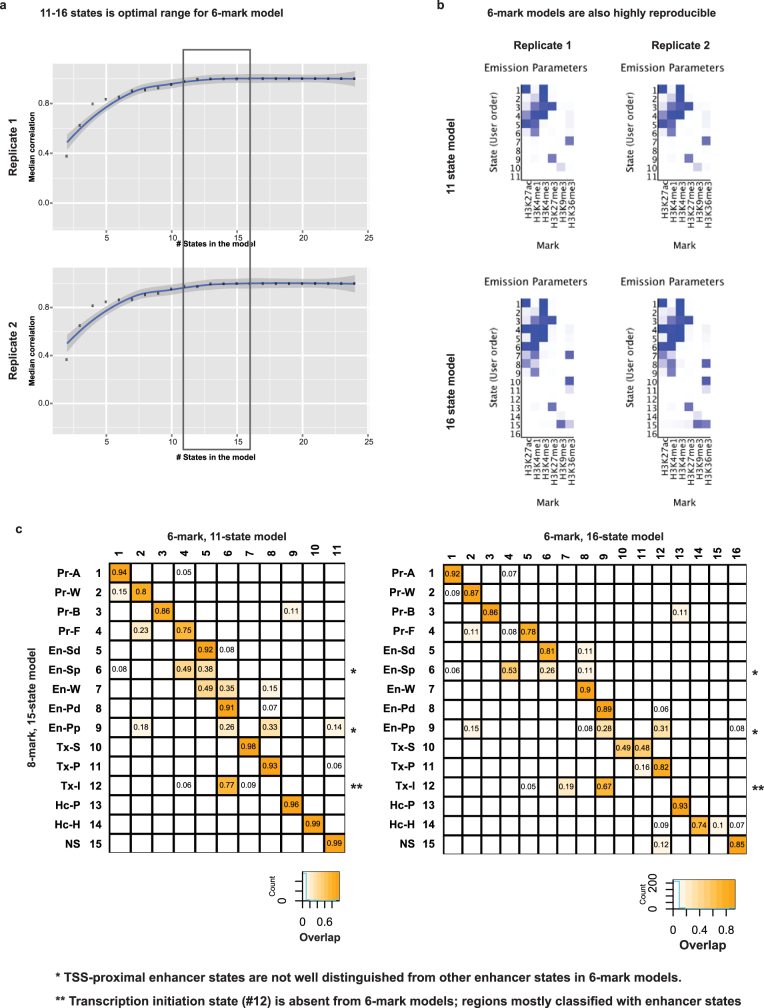
Comparing eight-mark ChromHMM model with six-mark models. **a**, Median correlations of the 24 states in the full model (*y*-axis) with its best matching state in each simpler model (*x*-axis). The box indicates that a value close to the maximum is already reached with an 11-state model, and a value virtually equal to the maximum is obtained with a 16-state model. Shaded area represents confidence intervals of the smoothing line obtained using stat_smooth() of ggplot2 (using default parameters, default method is LOESS). **b**, Emission probabilities for each histone modification in each state, as defined by ChromHMM, for both replicates (11-state model on top, and 16-state model at the bottom). **c**, Overlap of regions in each of the eight-mark 15-state models with the regions classified by the 11- and 16-state models using only 6 marks. Major differences are indicated by asterisks and explained below.

To leverage the chromatin state information captured by combinatorial patterns of histone modifications, we used ChromHMM^16^, which derived a 15-state model that shows near-perfect consistency between biological replicates and general agreement with previously published models^10,13,16^ (Fig. 2a, Extended Data Fig. 6; Methods). We segmented the genome for each tissue-stage with the full complement of eight histone modifications (*n* = 66 tissue-stages), excluding E10.5 to ensure a consistent approach (Extended Data Fig. 7). Each state was assigned a descriptive label based on its similarity to known chromatin signatures^5,13,17^, and genomic distribution (Extended Data Fig. 6i). The resulting chromatin state maps allow the visualization of multiple functional predictions across a range of tissues and stages (Fig. 2b).

**Extended Data Fig. 8:**
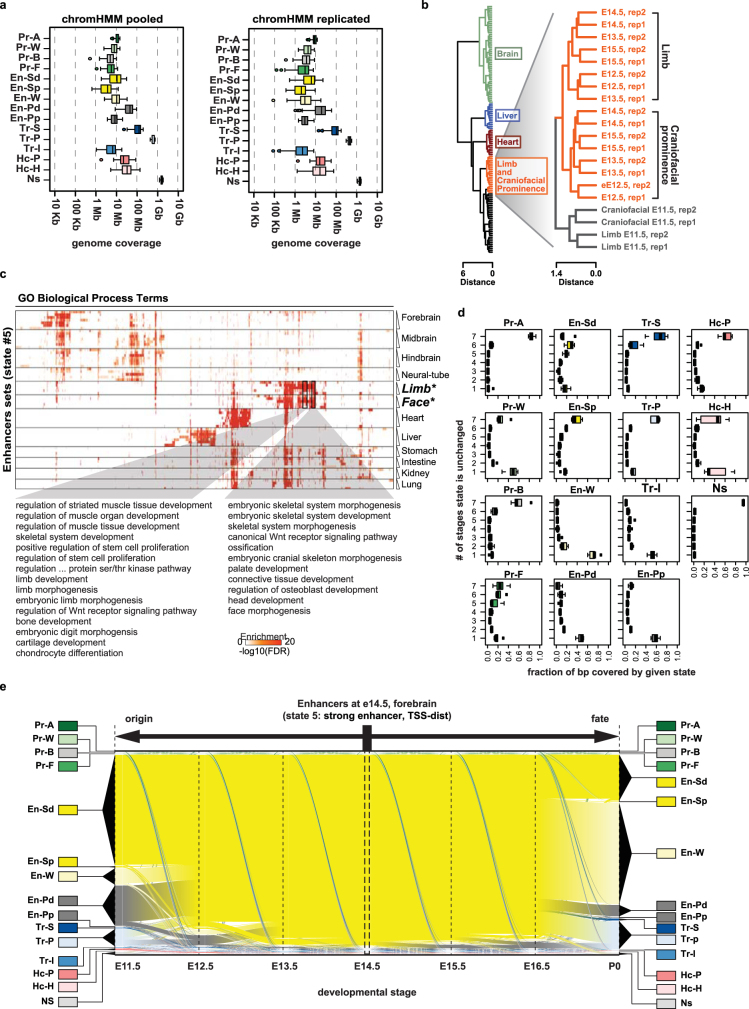
Chromatin state developmental dynamics. **a**, Enrichment of accessible chromatin within regions segmented into different chromatin states. Left, values for a set of ChromHMM annotations made using ChIP–seq data pooled from both biological replicates. Right, values for a more conservative set of ChromHMM annotations including only those regions annotated in the same state independently in both biological replicates. *n* = 66 tissue-stages per box. **b**, Hierarchical relationships among strong enhancers (state no. 5) in different tissues during development (clustering according to binary distance, Ward’s method). This analysis revealed a strong relationship between limb and facial tissue, also observed in clustering of specific histone modifications (Extended Data Fig. 4a), and further supporting the hypothesis of that facial structures and limbs have a common developmental origin^92,93^. **c**, Enrichment of functional terms (*x*-axis, GO biological processes, *P* values from GREAT binomial test; FDR is Benjamini–Hochberg corrected *q* value) for the sets of strong enhancers (state no. 5) across each tissue-stage (*y*-axis). Sample sizes provided in Supplementary Table 5. The terms were hierarchically clustered (average linkage) according to Pearson’s correlation. A subset of the terms highly enriched in both limb and face is listed below the main heatmap. **d**, Fraction of bases (*x*-axis) annotated in the indicated state consistently in up to seven stages sampled (*y*-axis). Only tissues sampled at seven stages are shown here (*n* = 5). **e**, Sankey diagram showing the origin and fate of all genomic intervals classified as TSS-distal strong enhancers (state no. 5) in E14.5 forebrain. The chromatin state classification of these regions was tracked across the available developmental stages, and the relative genomic coverage of each chromatin state at each transition is plotted. The thickness of each colour (*y*-axis) indicates the coverage of each state.

The 15 chromatin states fit into four broad functional classes: promoter, enhancer, transcriptional, and heterochromatin states. As expected, promoter states show the highest average levels of chromatin accessibility, followed by enhancer, transcriptional, and heterochromatin (Fig. 2c). In total, about 33% of the genome shows a reproducible chromatin signature characteristic of one of these four functional classes in at least one tissue-stage. In this calculation we required that a region be called in the same state in both biological replicates, and we excluded states 15 (‘no signal’) and 11 (‘permissive’), which covered large swaths of the genome (Fig. 2d, Extended Data Fig. 8a). This does not necessarily imply that 33% of the genome sequence is functional during development, but rather that 33% of the genome sequence is mappable and packaged in chromatin with a reproducible signature in at least one tissue-stage profiled here. These chromatin signatures often reflect transcriptional and/or regulatory activity, but the underlying sequences may not be under negative selection^18^.

**Extended Data Fig. 9:**
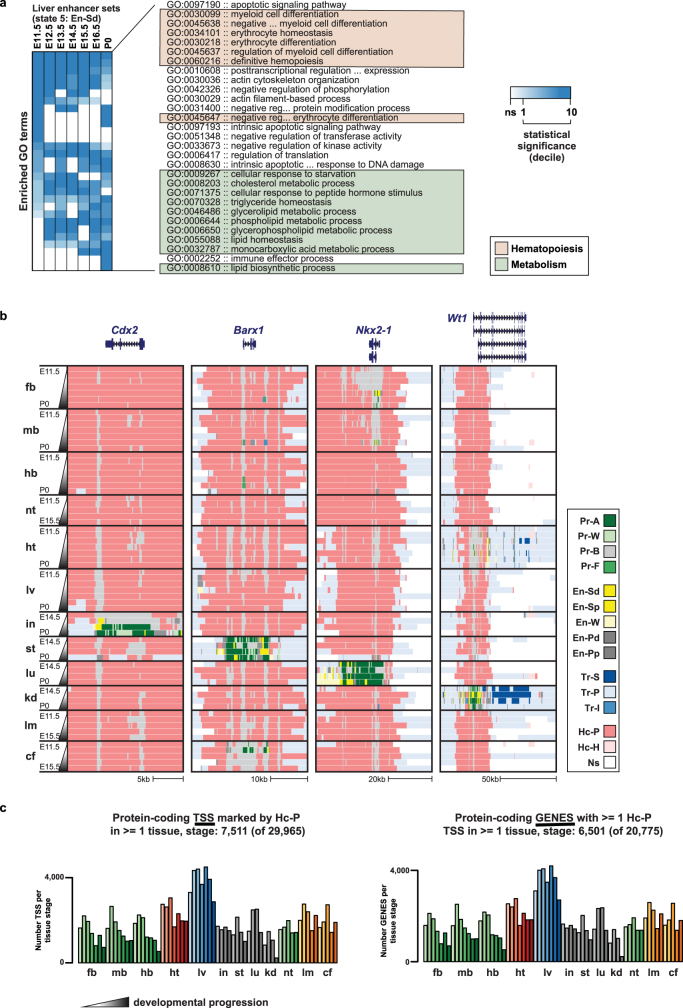
Chromatin state dynamics and signature of PcG repression at key regulators. **a**, The most enriched biological processes (GO terms) for genes near putative liver enhancers (*n* = 4,595). The significantly enriched terms for each stage were identified and divided into deciles (based on statistical significance). The ten most enriched terms for each stage were then grouped together and hierarchically clustered. Genes involved in either haematopoiesis or metabolic processes are colour-coded, as indicated. *P* values from binomial test (GREAT); FDR is Benjamini–Hochberg corrected *q* value. **b**, Genome browser views showing tissue-restricted activity patterns at *Cdx2* (chr5: 147,294,550–147,313,599; mm10), *Barx1* (chr13: 48,649,148–48,680,395; mm10), *Nkx2-1* (chr12: 56,507,647–56,560,509; mm10), and *Wt1* (chr2: 105,097,427–105,200,306). **c**, Left, the number of TSSs marked by Hc-P in each tissue-stage. Right, the number of genes with at least one annotated TSS marked by Hc-P in each tissue-stage.

The breadth of data collected here enabled us to characterize the spatial and temporal dynamics of chromatin states. On average, about 1.2% of the genome differs in chromatin state between tissues at the same stage (mean 1.2%, 31.3 Mb; range 1.0–4.0%, 26.8–109.1 Mb). Enhancer states are most variable between tissues, consistent with the role of enhancers in defining tissue and cell identity (Fig. 2e, Extended Data Fig. 8b–e). Indeed, hierarchical clustering based on strong enhancers alone (that is, state 5) distinguished tissues and identified similarities in developmental origin (Extended Data Fig. 8b, c). Within a given tissue, about 1.3% of the genome differs in chromatin state between adjacent developmental stages (mean 1.3%, 36.6 Mb; range 0.03–3.01%, 9.4–82.1 Mb). Enhancer states are most variable, although poised or weak enhancer states are more variable than strong enhancer states (Fig. 2e). Nonetheless, temporal changes in strong enhancer states can capture important developmental processes such as the transition of fetal liver function from haematopoiesis to metabolism (Extended Data Fig. 9a).

**Extended Data Fig. 10:**
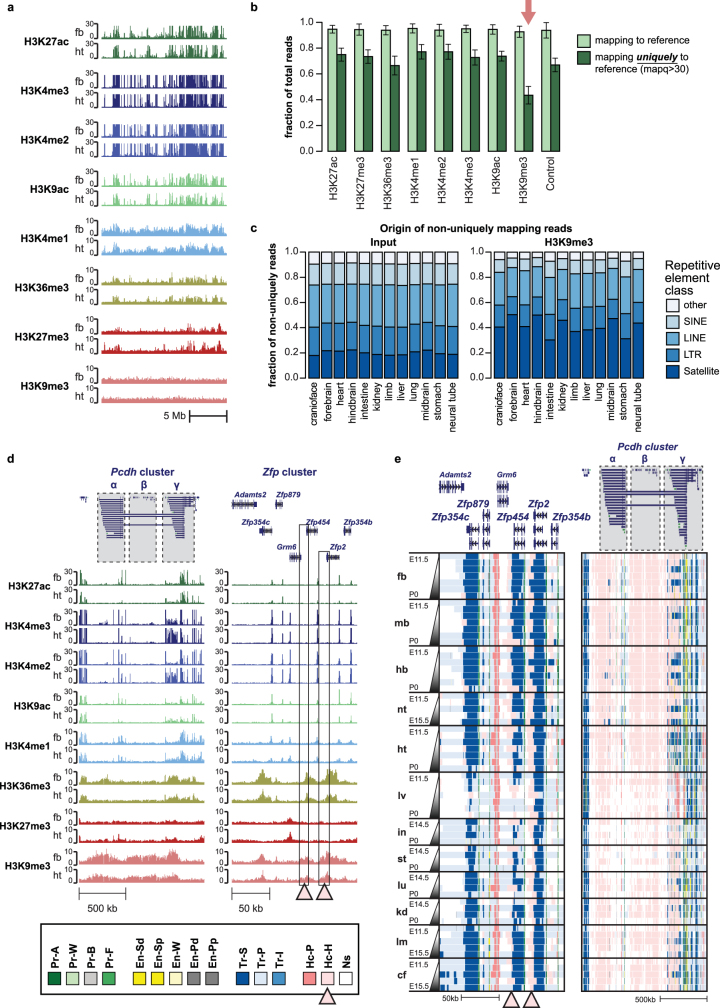
H3K9me3 heterochromatin. **a**, Genome browser view showing a large region of chromosome 15 (chr15: 87,165,311–104,043,685; mm10). Signal tracks (fold enrichment over input) are shown for all marks. H3K9me3 looks relatively flat, unlike the other marks. We find very few regions of strong H3K9me3 enrichment outside repetitive elements, consistent with previous reports of H3K9me3 distribution in primary tissues^26^. Data shown here and in **d** are from E15.5. **b**, The fraction of total sequencing reads that map to the reference genome (light green), and that map uniquely to the reference genome (mapq ≥30; dark green). *y*-axis is the mean for all ChIP libraries reported here separated by mark (*n* = 72 for all marks except for H3K4me2 and H3K9ac where *n* = 66), and error bars represent s.d. Control bars represent ChIP input libraries (no IP step). All marks and input have a high mapping rate (mean >90%), but H3K9me3 has a markedly low rate of unique mapping, suggesting that this modification is specifically enriched in non-unique (that is, repetitive) genomic regions. **c**, Stacked bar plots show the type of repetitive elements from which the non-uniquely mapping reads from **b** are likely to originate. H3K9me3 reads are highly enriched in satellite repeats relative to the input controls. **d**, Genome browser view of ChIP–seq fold enrichment tracks at *Pchd* (chr18: 36,720,767–38,058,585; mm10) and *Zfp454* (chr11: 50,774,724–50,939,391; mm10) shows significant H3K9me3 enrichment (state 14) during development. The 3′ UTRs of Zfp genes marked by H3K9me3 (reported previously^28^) are indicated by pink arrowheads. **e**, As in **d**, but showing chromatin states across these regions.

**Extended Data Fig. 11:**
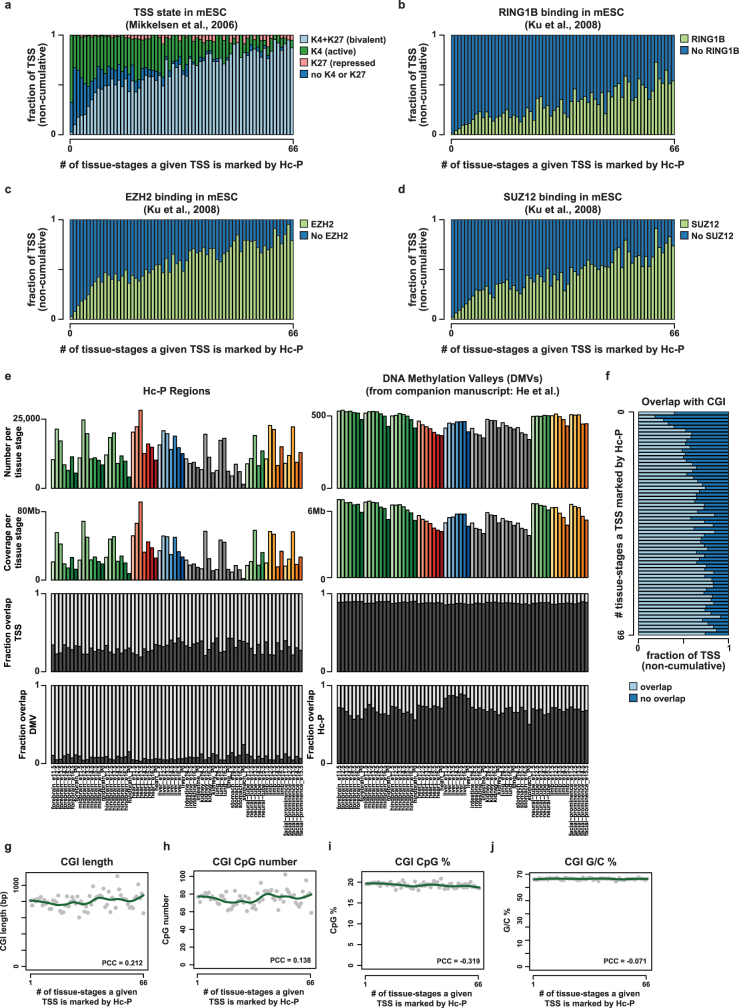
Properties of putative PcG target genes. **a**, TSSs are binned together according to the number of tissue-stages in which they are marked by Hc-P (0–66, *x*-axis). For each bin, the fraction of TSSs that are K4 + K27 (bivalent), K27 (repressed), K4 (active), or has no K4 or K27 in mouse ES cells is plotted, as reported previously^29^. **b**–**d**, Similar schema to **a**, but plotting the fraction of TSSs bound by RING1B (PRC1 component), EZH2 (PRC2 component), or SUZ12 (PRC2 component) in mouse ES cells, as previously reported^30^. **e**, Comparison of Hc-P regions as reported here and DMVs from ref. ^7^. Left, metrics related to regions annotated as Hc-P in each tissue-stage (*x*-axis). From top to bottom: number of Hc-P regions in each tissue-stage; coverage of Hc-P in each tissue-stage; fraction of Hc-P regions that overlap a TSS; fraction of Hc-P regions that overlap a DMV. Right, metrics related to regions annotated as DMVs in each tissue-stage (*x*-axis). From top to bottom: number of DMVs in each tissue-stage; coverage of DMVs in each tissue-stage; fraction of DMV regions that overlap a TSS; fraction of DMV regions that overlap a Hc-P region. **f**, Schema as in **a**–**d**, but with axes switched. For each bin, the fraction of TSSs that overlap a CGI is plotted on the *x*-axis. **g**–**j**, The following properties of CGIs that overlapped Hc-P TSSs are plotted (left to right): CGI length; CpG number; CPG percentage; GC percentage. None of these properties is strongly correlated with the number of tissue-stages in which a given TSS is marked by Hc-P (*x*-axis), supporting the role of factors other than CGIs in recruiting or excluding PcG at target promoters in a tissue- and/or stage-restricted fashion^94,95^. Green line shows LOESS smooth curve, span 0.25 and degree 1.

**Extended Data Fig. 12:**
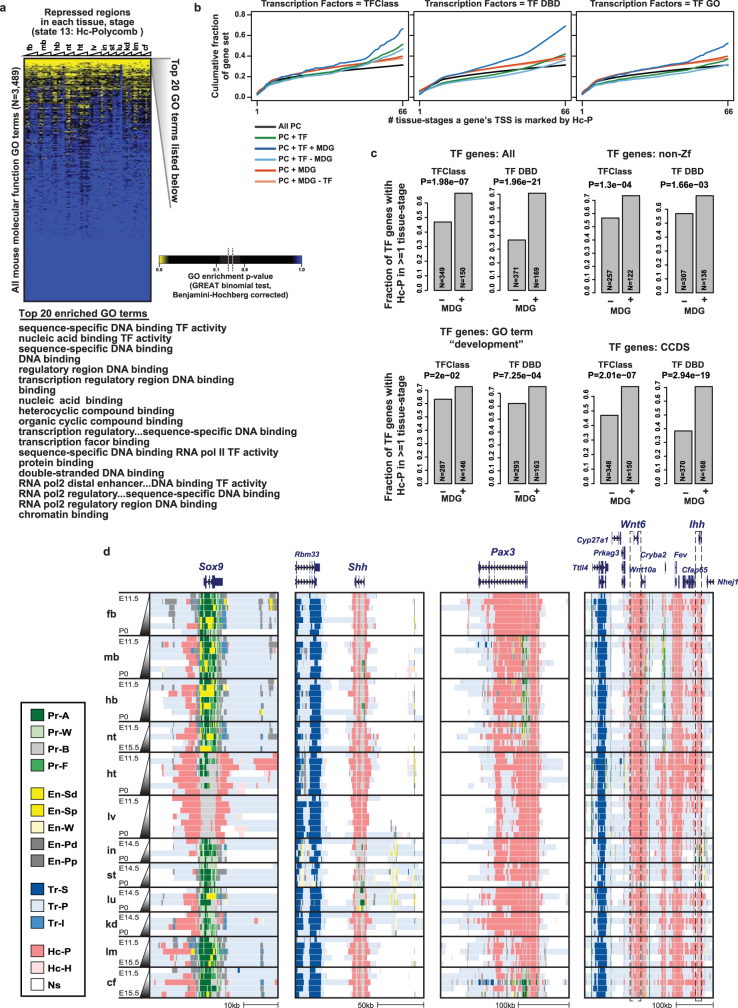
Hc-P enrichment at disease-relevant TF genes. **a**, Enrichment of ‘molecular function’ GO terms in genes near repressed regions (state 13, Hc-P) as measured by GREAT binomial test with Benjamini–Hochberg correction. GO terms on the *y*-axis are ordered by average enrichment *P* value across all tissue-stages. The top 20 GO terms are listed below, and are all related to TF function. Number of regions for each tissue-stage shown in Extended Data Fig. 11e. **b**, Similar layout to Fig. 2f. The fractions of six gene sets that show evidence of PcG repression are plotted: 1) all protein-coding genes (black line); 2) the subset of protein-coding genes that code for TFs (green line); 3) the subset of protein-coding genes that code for TFs and underlie human Mendelian diseases (dark blue line); 4) the subset of protein-coding genes that code for TFs but do not underlie human Mendelian diseases (light blue line); 5) the subset of protein-coding genes that underlie human Mendelian diseases; 6) the subset of protein-coding genes that underlie human Mendelian diseases but are not TFs. The origin of the TF super-sets is indicated on top of each sub-panel, from left to right: the TFClass database, the DBD database, and genes associated with a GO term containing the phrase ‘TF’. **c**, *P* values from *χ*^2^ test of independence between PcG repression and Mendelian phenotype involvement. Different subsets of TF genes were used for this analysis, clockwise from top to bottom: All, all genes annotated as TF in the indicated database (TFClass or DBD); non-Zf, genes annotated as TF but not as zinc finger, to ensure that the enrichment for disease genes is not coming only from this large family of TFs; GO term development, genes with a GO term containing ‘development’, to show that the enrichment for disease genes exists even amongst TFs that are all likely to have a role in development; CCDS, genes with transcripts annotated by the consensus coding sequence (CCDS) project, representing high-confidence gene annotations in both the mouse and human genomes. Sample sizes shown over each bar. **d**, Patterns of PcG repression at *Sox9* (chr11: 112,766,260–112,803,708; mm10), *Shh* (chr5: 28,392,703–28,531,239; mm10), *Pax3* (chr1: 78,027,730–78,280,060; mm10), and *Wnt6/Ihh* (chr1: 74,643,751–74,987,517; mm10). This small but well-characterized set of genes is known to cause human congenital phenotypes when expressed ectopically during development^46,96^.

We found that the Polycomb-associated heterochromatin state (Hc-P, state 13) is prevalent at well-characterized regulators of tissue development^19–23^ (Fig. 2b, Extended Data Fig. 9b), while another heterochromatic state characterized by H3K9me3 is found mainly in repetitive sequence, as previously described^24–28^ (Extended Data Fig. 10). To more systematically examine the role of Polycomb-group (PcG) proteins during mouse development, we assembled a list of 6,501 putative PcG target genes with transcription start sites (TSSs) marked by Hc-P in at least one tissue-stage (Extended Data Figs. 9c, 11, Supplementary Tables 1, 2), many of which overlapped with DNA methylation valleys (DMVs) in the same tissue-stage^7^ (Extended Data Fig. 11e). Most of these genes are previously described targets of PcG (Extended Data Fig. 11a–d), but roughly one quarter (*n* = 1,786) have not been described as PcG targets in mouse^29–32^, and 400 have not been described in human or mouse^13^. Consistent with previous reports^29–31^, TFs are highly enriched among PcG targets (Extended Data Fig. 12a). Furthermore, we find that TFs with known human Mendelian phenotypes (Mendelian disease genes, MDGs) are even more likely than other TFs to be PcG targets (1.42-fold, *P* = 2 × 10^−7^ considering all TFs; 1.23-fold, *P* = 1.3 × 10^−4^ excluding zinc finger TFs; Fig. 2f, g, Extended Data Fig. 12b–d). These data suggest that PcG-mediated repression has an essential and pervasive role in silencing key regulators outside their normal expression domains and point to failed repression as a potential disease mechanism for further exploration.

## Catalogue of regulatory sequences

**Fig. 3:**
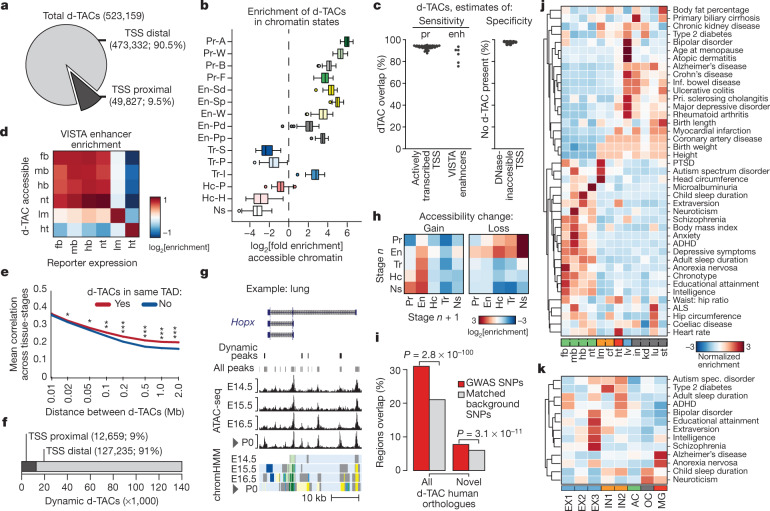
An expansive catalogue of regulatory sequences in mouse fetal development. **a**, Number of TSS-proximal and TSS-distal d-TACs. **b**, Enrichment of accessible chromatin within different chromatin states (*n* = 66 tissue-stages). **c**, Estimates of d-TAC catalogue sensitivity (left) and specificity (right). Six tissue-stages plotted for enhancers based on VISTA data availability (E11.5 forebrain, midbrain, hindbrain, limb, heart and neural tube). Eighteen tissue-stages plotted for DNase-inaccessible TSS based on matched DNase data available through the ENCODE portal. pr, promoter; enh, enhancer. **d**, Enrichment for elements that direct tissue-restricted reporter expression within d-TACs accessible in the corresponding tissue. **e**, Correlation of ATAC–seq signal across tissue-stages plotted as a function of genomic distance between d-TACs (*n* = 523,159). d-TACs are divided according to whether they are the same TAD (red line) or not (blue line). Two-sided Wilcoxon signed rank test, left to right: *P* = 0.04, 0.02, 2 × 10^−3^, 2 × 10^−4^, 1 × 10^−4^, 1 × 10^−4^, 1 × 10^−4^, 1 × 10^−4^. **f**, Number of dynamic TSS-proximal and TSS-distal d-TACs. **g**, Dynamic d-TACs in lung at *Hopx* (chr5: 77,084,370–77,116,768; mm10), a marker of mature alveolar type I cells^59^. **h**, Chromatin state changes at dynamic d-TACs that gain (left) or lose (right) accessibility. Enrichment relative to coverage of each state in total d-TAC catalogue. **i**, Enrichment of genome-wide association study (GWAS) single nucleotide polymorphisms (SNPs) in d-TAC human orthologues compared to background set generated with SNPsnap^60^. Hypergeometric test (all *n* = 190,462; novel *n* = 20,891, not described in catalogues of accessible chromatin regions in human). **j**, Enrichment of GWAS signal for complex traits and diseases (*y*-axis) within human orthologues of TSS-distal d-TACs from specific tissues (*x*-axis) with polyTest^61^. For GWAS sample sizes, see Supplementary Table 11. Enrichment values plotted are −log_10_[polyTest *P* values] *z*-score normalized within studies. **k**, As in **j**, but with TSS-distal accessible chromatin regions from published forebrain single-nucleus ATAC–seq^38^. EX, excitatory neurons (sub-clusters 1, 2, 3); IN, inhibitory neurons (sub-clusters 1, 2); AC, astrocytes; OC, oligodendrocytes; MG, microglia.

**Extended Data Fig. 13:**
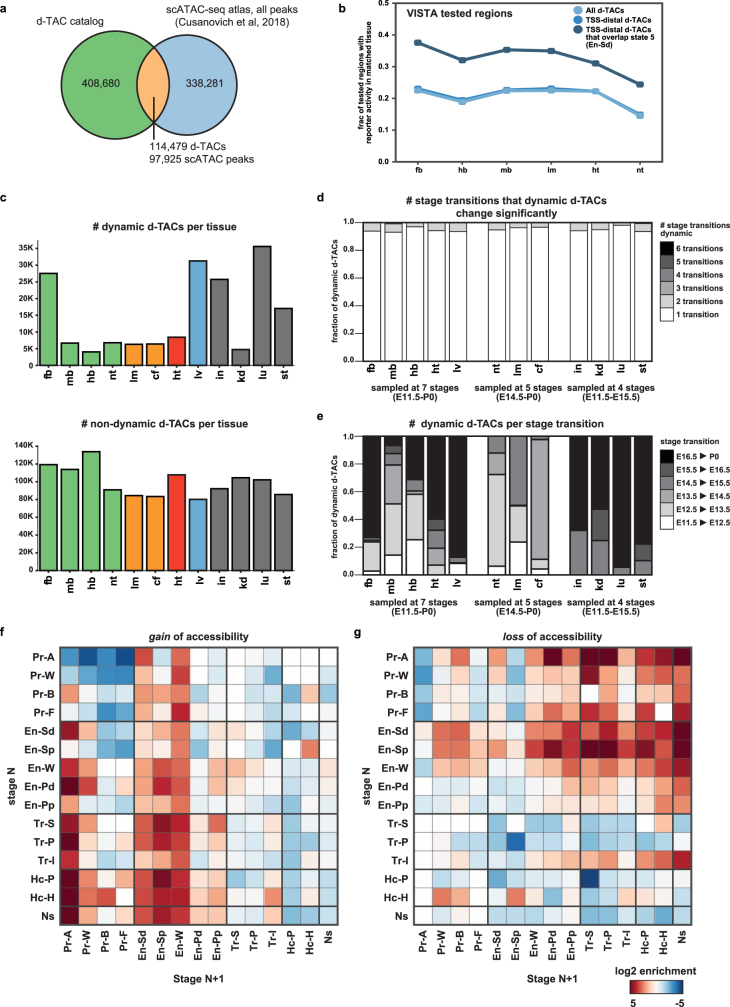
Dynamic d-TACs. **a**, Overlapping regions between our d-TAC catalogue and the adult single-cell ATAC–seq atlas from ref. ^33^. **b**, Fraction of tested d-TACs active in each tissue that exhibit positive reporter activity in the same tissue. This analysis was performed for three different sets of tissue-accessible d-TACs: all d-TACs, TSS-distal d-TACs, and TSS-distal d-TACs that overlap state 5 (strong TSS-distal enhancers). **c**, Top, number of dynamic d-TACs per tissue. Bottom, number of non-dynamic d-TACs per tissue. If a d-TAC was called as significantly dynamic at any stage transition within it a tissue it was labelled as dynamic; otherwise it was labelled as non-dynamic. **d**, Stacked bar plot shows the fraction of dynamic d-TACs in each tissue that are dynamic at one, two, three, four, five, or six stage transitions. **e**, The fraction of dynamic d-TACs within a tissue that undergo significant changes in accessibility at each stage transition. **f**, Similar schema to Fig. 3h but showing each chromatin state separately instead of as supersets. The heatmap shows the chromatin state changes that occur at dynamic d-TACs that gain accessibility at a given stage transition. Enrichment is relative to the coverage of each state in total d-TAC catalogue. **g**, As in **f**, but for d-TACs that lose accessibility at a given stage transition.

To build a catalogue of candidate regulatory sequences in mouse fetal development, we identified a non-overlapping set of 523,159 regions that were accessible in at least one tissue-stage, referred to below as developmental regions of transposase-accessible chromatin (d-TACs) (Fig. 3a, Supplementary Table 3). We note that this d-TAC catalogue is based only on the mouse tissue ATAC–seq data reported here, and is thus distinct from the ENCODE Registry of Candidate *cis*-Regulatory Elements (ccREs) (http://screen.encodeproject.org/), which incorporates data from other samples and assays^53^. Approximately 22% of d-TACs overlap with peaks from a single-cell ATAC–seq atlas of adult mouse tissues published while this manuscript was in revision^33^ (Extended Data Fig. 13a). We find that d-TACs are enriched in promoter and enhancer states, but generally depleted in states that characterize gene bodies, heterochromatin, and regions with no chromatin signature (Fig. 3b). Most d-TACs are distal to annotated TSSs, representing putative enhancers and other TSS-distal elements (90% of d-TACs are more than 1 kb from a TSS). Comparison with the VISTA database^34^ shows that about 20% of d-TACs tested show in vivo reporter activity in the corresponding tissue (Extended Data Fig. 13b), and 76–94% of in vivo validated enhancers are d-TACs in the corresponding tissue at E11.5 (VISTA reporter expression measured in E11.5 embryos; Fig. 3c, d).

To more directly assess the temporal dynamics of chromatin accessibility during development, we identified 139,894 dynamic d-TACs that exhibit a significant change in accessibility in at least one stage transition within a tissue (27% of all d-TACs; Fig. 3f, g, Extended Data Fig. 13c). Most dynamic d-TACs show a significant change at only one stage transition in this developmental window (Extended Data Fig. 13d, e), suggesting that these changes reflect enduring shifts in cell fate and/or composition rather than rapid on–off switches. Gain or loss of accessibility often corresponds to gain or loss of enhancer chromatin states, respectively (Fig. 3h, Extended Data Fig. 13f, g). In addition, d-TACs close to each other in the genome are more likely to have correlated activity across tissue-stages (Fig. 3e, Supplementary Table 3), particularly when located in the same topologically associating domain (TAD)^35^.

Catalogues of candidate regulatory sequences can provide valuable resources for the interpretation of non-coding genetic variation linked to disease^33,36,37^. Thus, we investigated whether our d-TAC catalogue could provide insights into the genetics of human disease. We first identified putative human orthologues of our mouse d-TACs (Supplementary Table 4). Approximately 89% (169,571 of 190,462) of these human sequences have been annotated as accessible chromatin in human cells^9,36^, suggesting that they have conserved function. We found that phenotype-associated genetic variation is enriched in the putative human orthologues of mouse d-TACs, including at regions not previously annotated as accessible in human^9,36^ (Fig. 3i). Moreover, these enrichments show patterns of tissue specificity which may link diseases to tissue-dependent and possibly fetal regulatory programs (Fig. 3j). However, these patterns can be difficult to interpret, in part because the ATAC–seq data come from heterogeneous tissues. Our group recently published single-nucleus ATAC–seq of the mouse forebrain^38^, allowing us to further deconvolute several enrichments into specific cell types in this tissue (Fig. 3k). Analysis of human orthologues of mouse enhancer predictions based on DNA methylation (feDMRs) has produced similar results^7^.

## Developmental enhancer dynamics

**Fig. 4:**
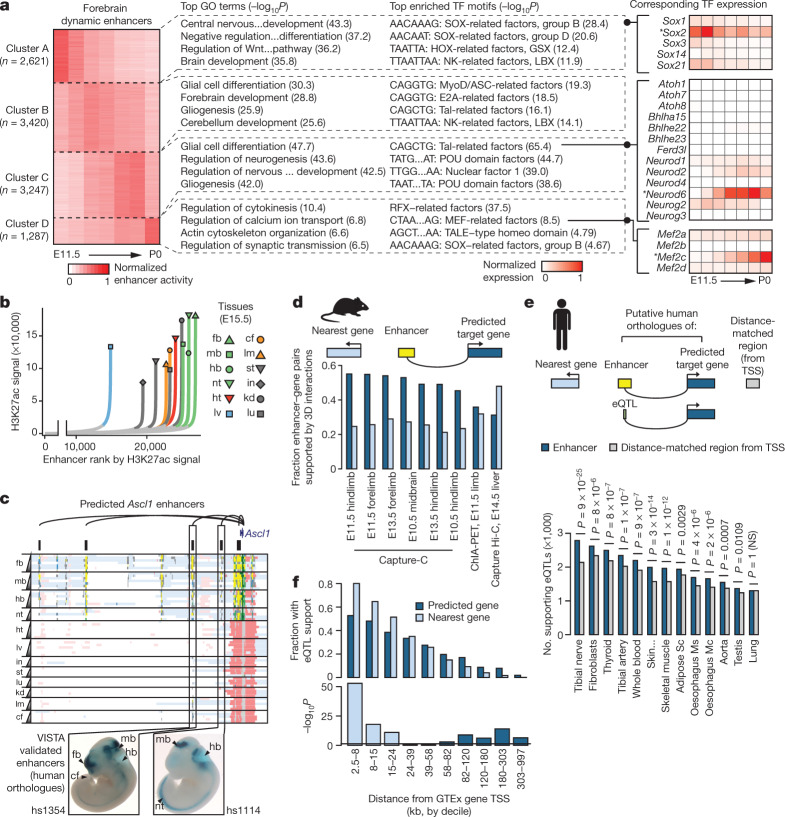
Developmental enhancer dynamics reveal key regulators and link enhancers to target genes. **a**, *k*-means clustering (*k* = 4) of dynamic forebrain enhancers based on H3K27ac signal. The top enriched biological process GO terms by GREAT are plotted next to each cluster, and the top sequence motifs enriched in each cluster are plotted next to the GO terms. Some motifs and GO terms are abbreviated to fit. Heatmap to the right shows normalized gene expression for related TFs that potentially correspond to the motifs indicated by black circles. *TFs mentioned in text. **b**, The distribution of H3K27ac signal (read counts) across all enhancers identified in each tissue at E15.5. Super-enhancers show exceptionally high signal (coloured lines). **c**, Predicted enhancers of *Ascl1* (chr10: 87,301,848–87,515,210; mm10). Enhancers with human orthologues validated by in vivo reporter assays are shown below main panel. Arrowheads, tissues with reproducible staining. **d**, Enhancer target genes supported by published chromatin interaction data obtained using Capture-C^48^, ChIA–PET^49^ and Capture Hi-C^50^. The liver Capture Hi-C data set contains by far the most interactions (about 600,000), which may explain why the nearest gene assumption works in this data set only. **e**, Number of eQTLs (*y*-axis) supporting human orthologues of enhancer target gene predictions relative to TSS distance matched regions. Two-sided Fisher’s exact test. **f**, Genes binned into deciles by distance between enhancer and putative target gene (*n* = 13,873 pairs). Lower plot shows −log_10_(*P*) by two-sided Fisher’s exact test. Horizontal line indicates *P* = 0.05. NS, not significant.

**Extended Data Fig. 14:**
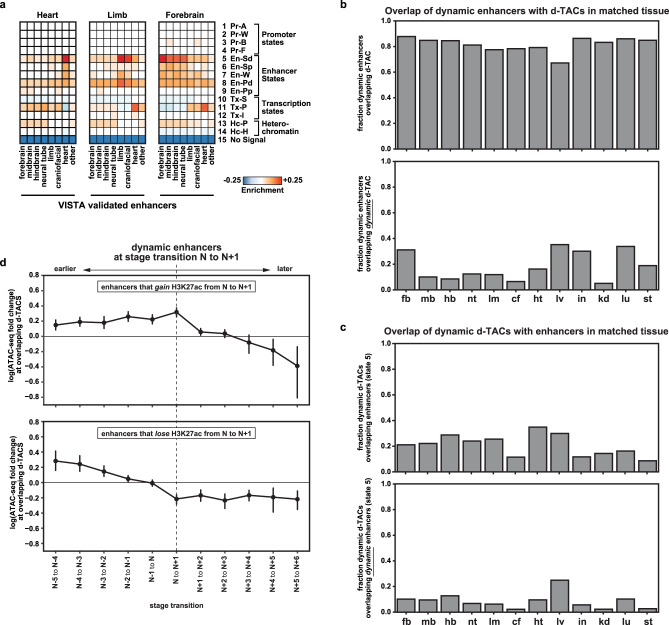
Chromatin state-based enhancers. **a**, Tissue-specific enrichments of VISTA enhancers for different chromatin states in E11.5 heart, limb and forebrain. **b**, Top, fraction of dynamic enhancers in each tissue (based on H3K27ac) that overlap d-TACs accessible in the matching tissue. Bottom, fraction of dynamic enhancers in each tissue that overlap d-TACs that were also called as dynamic by ATAC–seq in the matching tissue. **c**, Top, fraction of dynamic d-TACs in each tissue that overlap enhancers called by ChromHMM (state 5) in the matching tissue. Bottom, fraction of dynamic d-TACs in each tissue that overlap dynamic enhancers called with H3K27ac in the matching tissue. Each point represents one tissue-stage (*n* = 66). **d**, Top, dynamic enhancers that gain H3K27ac at a given stage transition *n* to *n* + 1. Lines show the log_2_ fold change in ATAC–seq signal within d-TACs that overlap those dynamic enhancers at various stage transitions. Dynamic enhancers that gain H3K27ac at a given stage transition tend to gain accessibility as measured by ATAC–seq either at or before the stage transition in question (sometimes preceding H3K27ac gain by as much as five stage transitions). Mean and s.d., filled circles and vertical lines, respectively. Bottom, dynamic enhancers that lose H3K27ac at a given stage transition *n* to *n* + 1. Dynamic enhancers that lose H3K27ac at a given stage transition tend to lose accessibility as measured by ATAC–seq either at or after the stage transition in question (sometimes proceeding H3K27ac loss by as much as five stage transitions). The number of stage comparisons for each offset is: ±0 *n* = 54, ±1 *n* = 42, ±2 *n* = 30, ±3 *n* = 18, ±4 *n* = 10, ±5 *n* = 5.

**Extended Data Fig. 15:**
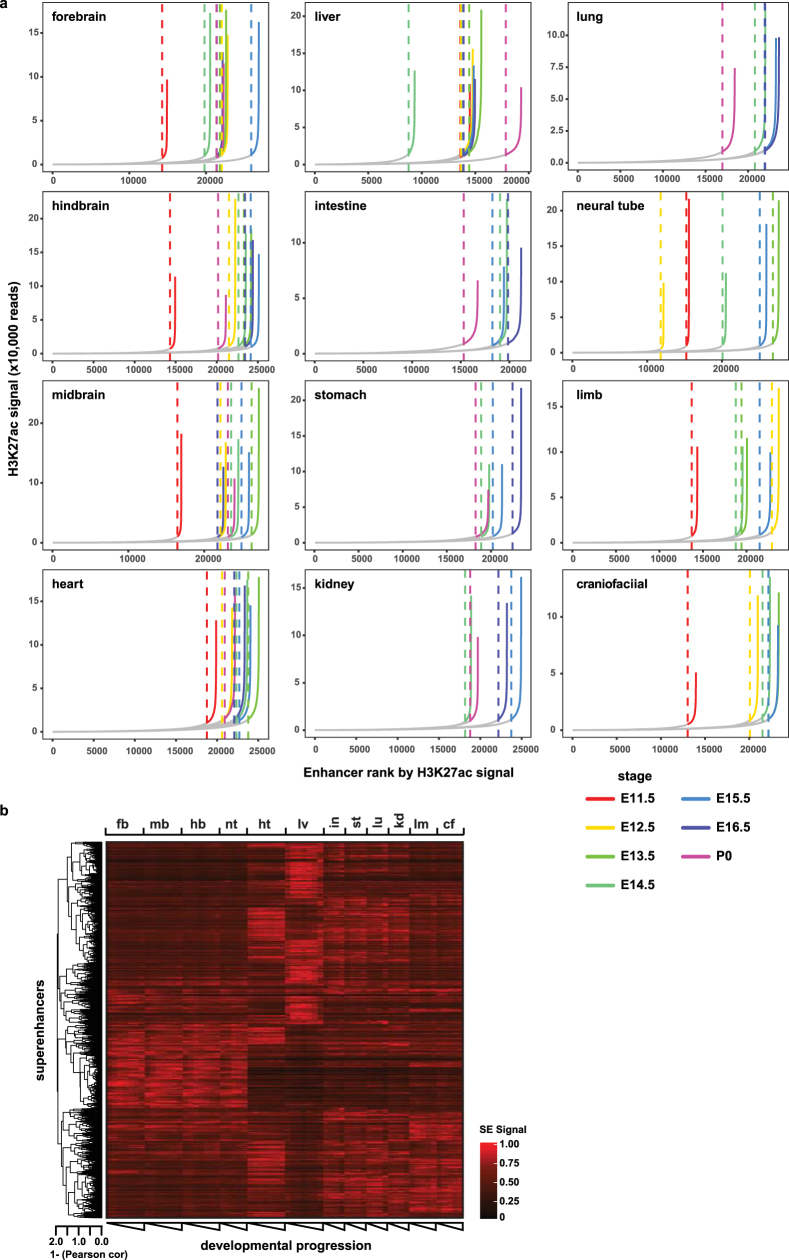
Super-enhancers. **a**, Distribution of the H3K27ac signal (read counts) across all enhancers identified in each tissue. Within each tissue, different stages are plotted as separate lines. A subset of the enhancers (super-enhancers) show exceptionally high levels of signal, as represented by coloured lines. **b**, Heatmap shows the normalized H3K27ac signal for all super-enhancers merged across tissues and stages (*n* = 4,833). The rows are hierarchically clustered according to Pearson’s correlation distance.

Given the important role of enhancers in directing gene expression, we focused on dynamic enhancers as a window into the developmental processes and regulatory factors in each tissue. We identified a high-confidence set of candidate enhancers marked by the strong TSS-distal enhancer state (Extended Data Fig. 14a, Supplementary Table 5), and identified ‘dynamic’ candidate enhancers for which the H3K27ac-based activity score changed from stage-to-stage^39^ (Methods). Most dynamic enhancers overlap d-TACs (67–88%, median 84%), but fewer overlap dynamic d-TACs (5–35%, median 14%; Extended Data Fig. 14b, c). This may reflect temporal differences in H3K27ac and accessibility dynamics (Extended Data Fig. 14d). We also used our H3K27ac data to identify ‘super-enhancers’, which are known to mark key regulators and have important roles in development^40^ (Fig. 4b, Extended Data Fig. 15, Supplementary Table 6).

**Extended Data Fig. 16:**
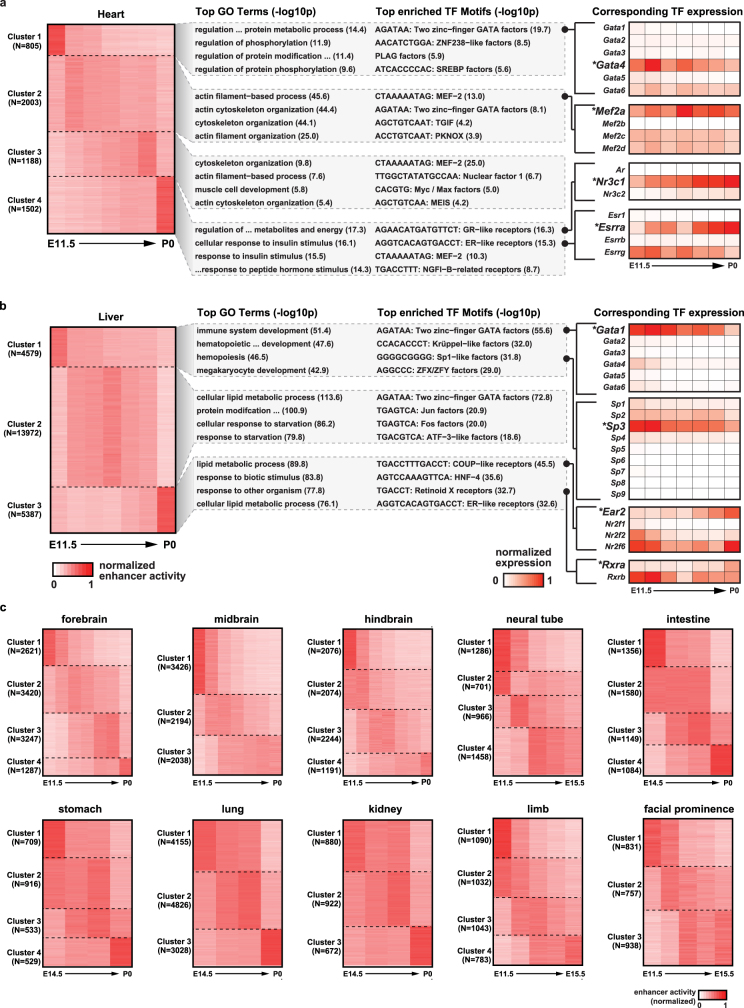
Dynamic chromatin state-based enhancers. **a**, Same layout as Fig. 4a, but for dynamic enhancers in heart. **b**, As in **a**, but for liver. GO biological process enrichment determined by GREAT^69^. Motif enrichment *P* values calculated by two-sided Fisher’s exact test. **c**, *k*-means clustering of dynamic enhancers for all other tissues based on H3K27ac signal at available stages from E11.5 to P0. The number of clusters within each tissue, and the number of dynamic enhancers within each cluster, are indicated to the left of each heatmap. Corresponding GO enrichment and motifs are provided in Supplementary Table 7. Sample sizes are indicated the left of each heatmap.

To gain deeper insights into the processes and regulatory factors in each tissue we clustered dynamic candidate enhancers and examined Gene Ontology (GO) terms associated with nearby genes, enrichment for TF binding motifs, and expression patterns of TFs corresponding to those motifs (Fig. 4a, Extended Data Fig. 16, Supplementary Table 7). Considering the forebrain as an example, we found four predominant clusters (labelled A–D, Fig. 4a). Cluster A represents enhancers that are active early, associated with GO terms related to general CNS development, and enriched for motifs that probably reflect the role of SOX2 in early brain development^41,42^. Clusters B and C contain enhancers that are most active in middle stages, associated with neurogenesis and gliogenesis, and enriched for motifs that probably reflect the role of NEUROD6 in neurogenesis during mid-to-late gestation^43^. Cluster D includes enhancers that are active late, associated with synaptic function, and enriched for motifs that support a role for MEF2C in synapse formation^44^.

**Extended Data Fig. 17:**
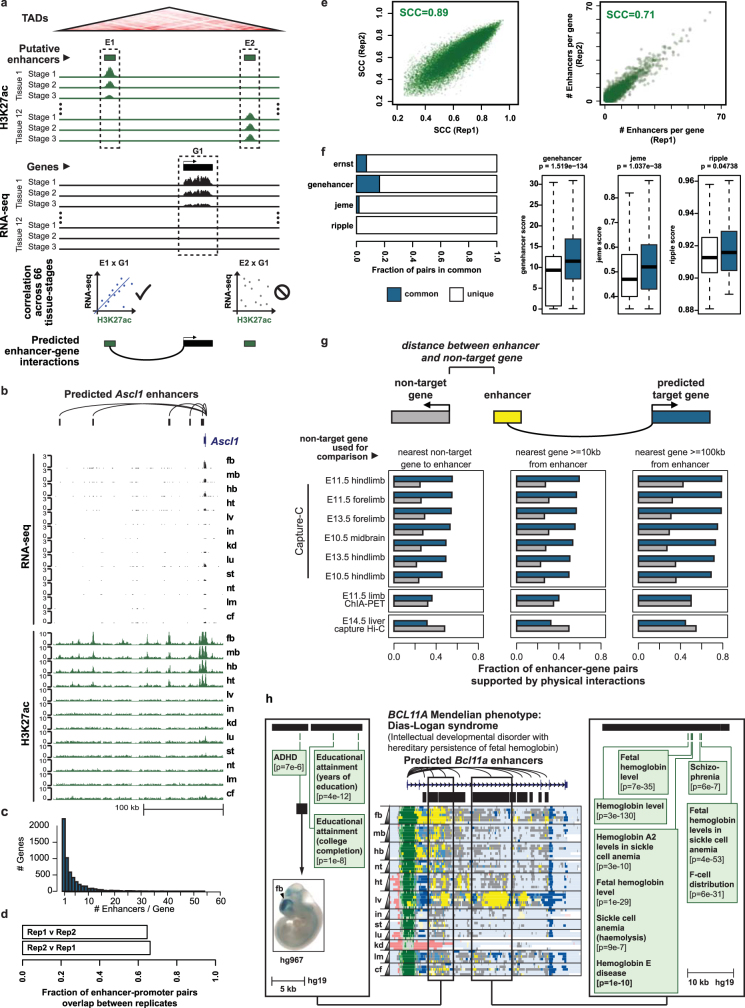
Enhancer target gene predictions. **a**, Schematic of the approach to assign enhancers to target genes. **b**, Genome browser view showing the *Ascl1* locus, as in Fig. 4c, but showing ChIP–seq fold enrichment tracks instead of chromatin states. **c**, Histogram of the number of enhancers per gene. **d**, For each replicate, the fraction of putative enhancers assigned to the same gene using data from the other available replicate. **e**, Scatter plots showing reproducibility of enhancer–gene maps as measured by correlation between enhancer–gene pairs (left; *n* = 21,141 pairs), and the number of enhancers per gene (right; *n* = 5,611 genes). **f**, Left, fraction of enhancer–gene associations that overlap interactions previously reported in ref. ^6^ (*n* = 907/12,655), GeneHancer^81^ (*n* = 2,067/12,546), JEME^82^ (662/36,007), and RIPPLE^83^ (31/37,541). The global level of overlap is low, perhaps in part owing to the different sample types used to predict these interactions. Right, distribution of scores for the unique and overlapping pairs in GeneHancer, JEME and RIPPLE, respectively. Where predictions from those reports overlap with ours, their scores are significantly higher. *P* values calculated using two-sided Mann–Whitney *U* test. **g**, As in Fig. 4d, this plot shows that enhancer–gene interactions identified by this correlative approach are generally more likely to be supported by chromatin interaction data than associations derived by a nearest gene approach. To ensure that this was not due to an artefact of the chromatin capture technologies being unable to detect short-range interactions, we used different distance cutoffs (10 kb, 100 kb) to define the ‘nearest’ non-target gene. **h**, The *Bcl11a* locus (chr11: 24,044,043–24,197,927; mm10) provides an interesting case in which genetic variation in enhancers regulating a pleiotropic Mendelian disease gene may contribute to tissue-restricted phenotypes with lower penetrance. Boxes outline enhancer clusters with active chromatin signatures in the CNS (left) and liver (right), and which have validated activity in the CNS and erythroid lineage, respectively^34,40,97^ (mouse embryonic liver is a site of erythropoiesis). The subpanels on either side of the main browser view show regions of the human genome that correspond to either the CNS enhancer cluster (left, chr2: 60,752,530–60,767,198; hg19) or liver enhancer cluster (right, chr2: 60,711,940–60,741,118; hg19). Thick black bars on top represent orthologues of the predicted *Bcl11a* enhancers, and thin green bars below represent GWAS SNPs for the EMBL-EBI GWAS catalogue.

The dynamic activity observed across tissue-stages provided the opportunity to predict enhancer target genes using the correlation between gene expression (as measured by RNA-seq) and H3K27ac enrichment at candidate enhancers within the same TAD^45–47^ (Fig. 4c, Extended Data Fig. 17a–c, Supplementary Table 8). We derived independent target gene maps for each biological replicate comprising 31,964 and 32,734 enhancer–gene assignments, respectively, with an overlap of 21,141 used for downstream analyses (Extended Data Fig. 17d–f). This correlation-based map predicts experimentally determined enhancer–gene interactions^48–50^ with higher accuracy than assigning an enhancer to the nearest gene (Fig. 4d, Extended Data Fig. 17g). We further examined whether this map could be useful for predicting human enhancer–gene relationships (Extended Data Fig. 17h, Supplementary Table 9). We hypothesized that if our mouse predictions are applicable to human, we should see enrichment for human expression quantitative trait loci (eQTLs)^51^ that link the human orthologues of mouse enhancers to the predicted target gene(s) by genetic association. Indeed, across a variety of human tissues we see significant enrichment of eQTLs that link predicted target genes to candidate enhancers relative to regions equidistant from but on the other side of the target TSS (12 of 13 tissues with *P* ≤ 0.05, Fisher’s exact test; Fig. 4e), and relative to the nearest-gene approach when the distance between TSS and eQTL is larger than about 50 kb (Fig. 4f). This distance-dependent effect may reflect our choice to consider only the ‘strong TSS-distal enhancer’ state, as well as the fact that TSS-proximal eQTLs are more likely to tag causative variants in promoters, splice sites, or other non-enhancer elements.

## Enhancer validation in vivo

**Fig. 5:**
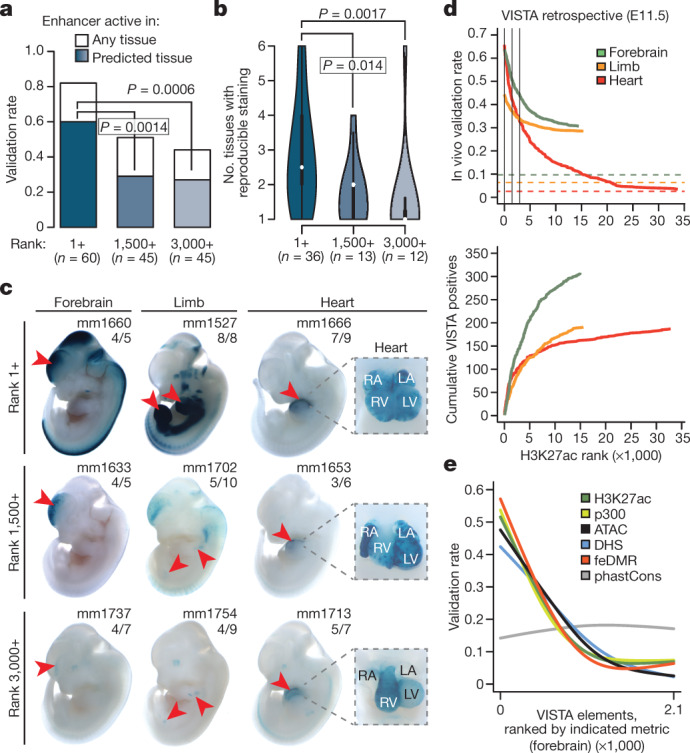
Systematic analysis of enhancer validation rate in vivo. **a**, Proportion of enhancers in each rank tier with reproducible staining in the expected tissue (blue) or any tissue (white). One-tailed Fisher’s exact test. **b**, Number of tissues with reproducible reporter expression, for all enhancers that validated in the expected tissue. One-tailed Mann–Whitney *U* test. White circles, median; black rectangles, IQR; whiskers, most extreme value within ±1.5 × IQR. **c**, Example enhancers from each tissue type and rank category that validated in the expected tissue. Representative transgenic E12.5 embryos show reporter expression (blue staining), along with the unique VISTA identifier and reproducibility (fraction of embryos with consistent staining). Far right, magnified images of heart (RA, right atrium; LA, left atrium; RV, right ventricle; LV, left ventricle). Red arrowheads, enhancer activity pattern. **d**, Retrospective analysis of 422, 299, and 414 elements in VISTA showing E11.5 activity in forebrain, limb or heart, respectively. Top, validation rate as a function of E11.5 H3K27ac rank. Horizontal dashed lines indicate estimated background validation rate for each tissue. Thin vertical lines mark the 1st, 1,500th, and 3,000th ranks. Bottom, cumulative number of positive enhancers as a function of H3K27ac rank. **e**, Enhancer validation rate across forebrain VISTA elements ranked with different genomic data sets (colours).

**Extended Data Fig. 18:**
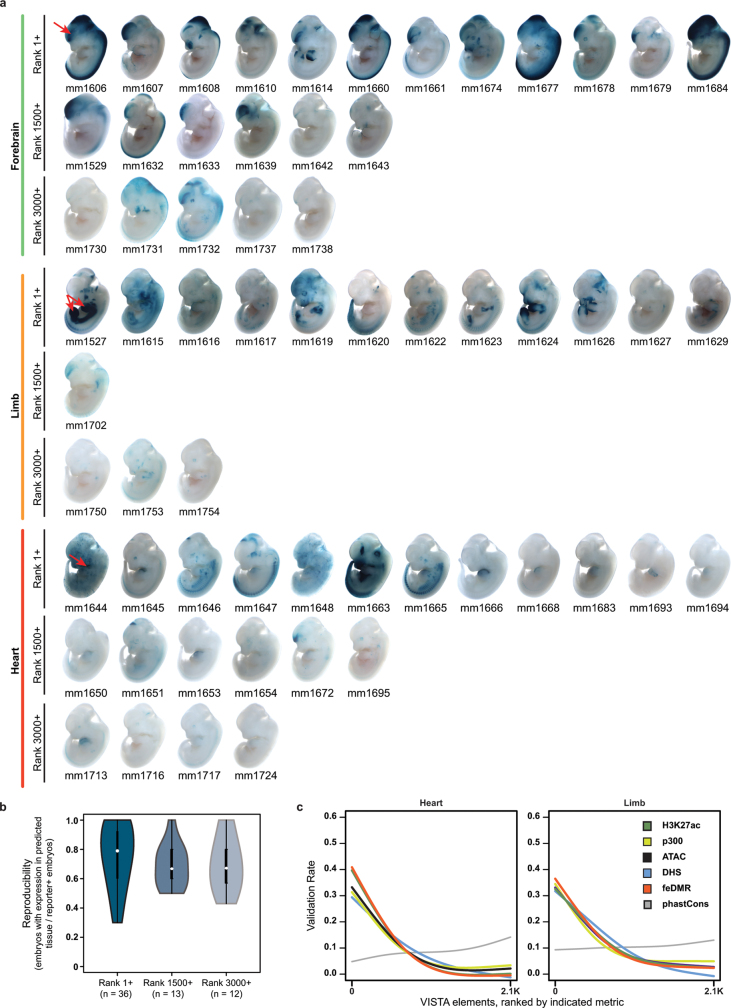
Transgenic validation results for predicted enhancers. **a**, Representative E12.5 transgenic embryos for each of the 61 enhancers that validated in the expected tissue (forebrain, limb, or heart). Reporter gene expression is indicated by blue staining, and enhancer names (mm numbers) are the unique identifiers from the VISTA Enhancer Browser^34^. Reproducibility for each enhancer is available in Supplementary Table 10 and through VISTA. Red arrows indicate forebrain, limbs, or heart. See also Supplementary Table 10 for results. **b**, Violin plots show transgenic enhancer assay reproducibility (that is, the percentage of embryos with reproducible activity) for different rank classes of tested elements. Only those enhancers that validated in the correct expected tissue are shown. Reproducibility differences between rank classes were not statistically significant (Mann–Whitney *U* test). Violin plots as in Fig. 5b, sample sizes shown below each violin. **c**, Same schema as in Fig. 5e, but for heart (left) and limb (right).

Histone modifications and chromatin accessibility are effective tools for identifying enhancers^5,39^, but the quantitative accuracy of these methods has not been well characterized. The level of H3K27ac enrichment can vary by orders of magnitude across peaks within a single data set. During previous studies^39^ we noticed that regions with stronger H3K27ac validated more frequently in transgenic reporter assays. To more systematically examine the relationship between H3K27ac signal and validation rate, we used transgenic mouse reporter assays^52^ to test 150 enhancers identified in tissues at E12.5, selected from three H3K27ac enrichment rank tiers: tier A (selected from ranks 1–85), tier B (ranks 1,500–1,550), and tier C (ranks 3,000–3,050) (Fig. 5a–c, Extended Data Fig. 18a, Supplementary Table 10). The full list of candidate enhancers from which these elements were chosen contains 35,955, 42,732, and 42,903 elements for forebrain, heart, and limb. About 60% of tier A elements displayed reporter expression in the expected tissue, compared to less than 30% from the two lower-rank tiers (Fig. 5a, *P* < 0.01, Fisher’s exact test). Tier A regions that validated in the expected tissue were also more likely to show activity in additional tissues (Fig. 5b, *P* < 0.05, Mann–Whitney *U* test), although we found no significant differences in overall reproducibility between tiers (Extended Data Fig. 18b). At all tiers, the validation rate is higher than background rates estimated from regions in the VISTA database that lack H3K27ac (heart 2.6%, limb 6.4% and forebrain 9.7%). Moreover, these background rates may overestimate the true genomic background because many VISTA elements were originally tested owing to evolutionary sequence conservation or epigenomic signatures that predict regulatory function.

Retrospective analysis of more than 2,000 regions assayed in vivo and catalogued in VISTA (assayed at E11.5) confirmed the trend described above across a much larger set of test elements (Fig. 5d, Supplementary Table 12). This larger set of elements also allowed us to evaluate other epigenomic data sets. Ranks based on p300 or H3K27ac ChIP–seq have the highest accuracy, followed closely by ATAC–seq and DNase hypersensitivity assays (Fig. 5e, Extended Data Fig. 18c). A combined score^54^ incorporating ChIP–seq, ATAC–seq, and DNA methylation as reported in an accompanying manuscript^7^ slightly outperforms any individual datatype. Taken together, these results demonstrate that loci with stronger enrichment for marks of enhancer activity such as H3K27ac are more likely to direct reporter expression in the expected tissue.

## Discussion

In summary, our results describe a multi-tiered compendium of functional annotations for the developmental mouse genome, including chromatin state maps for 72 distinct tissue-stages, an extensive catalogue of candidate regulatory sequences (many with dynamic temporal activity), enhancer target gene predictions, and a collection of transgenic reporter assays that demonstrates a strong relationship between H3K27ac signal and validation rate. The results of these reporter assays inform a key question in the field: what proportion of sequences with enhancer chromatin signatures truly function as enhancers in vivo? Surveys of chromatin state and chromatin accessibility in a single sample often predict enhancers numbering in the tens or even hundreds of thousands. However, the results of our in vivo reporter assays suggest that the validation rate of chromatin-based enhancer predictions decreases rapidly with rank based on H3K27ac level. While these results point to the uncertainty inherent in estimates of enhancer abundance, we do not think these estimates should be abandoned entirely. Definitive proof of an enhancer’s function (or lack thereof) requires more than reporter assays, and remains difficult to ascertain experimentally in a high-throughput manner. Ultimately, we think that our results highlight the importance of continued investigation into the molecular basis of enhancer function, as well as the predictive power of chromatin-based enhancer signatures.

Despite the broad scope of this study, we note some important limitations. First, there are multiple developmental tissues that were not surveyed here (for example, skeleton, gonads and pancreas). Second, as noted above, the tissues examined here are heterogeneous, and future efforts to examine the epigenomes of single cells during development will be critical to achieve a deeper understanding of developmental gene regulation. In addition, this study does not address sex-dependent aspects of development. Nonetheless, to our knowledge, the survey of fetal chromatin landscapes reported here is unprecedented in its breadth. Moreover, the developmental tissue panel examined here is the subject of complementary analyses focused on DNA methylation dynamics including methylation-aware enhancer predictions^7^, transcriptomic analysis including deconvolution of whole-tissue data into distinct cell types^98^, prediction of mammalian enhancers using evolutionarily conserved epigenetic patterns identified through massively parallel regulatory assays such as STARR-seq^55^, annotation studies focusing on genome evolution through the analysis of pseudogene complements across mouse strains^56^, identification of transcriptional waves mediated by tissue-stage-specific TFs^57^, and uncovering DNA motifs regulating histone modifications^58^. Given the key role of the mouse as a model system in biomedical research, we believe that these data and insights will be a valuable resource to the biomedical research community. All data sets, methods, and protocols are available at https://www.encodeproject.org/.

## Methods

### Tissue collection

All animal work was reviewed and approved by the Lawrence Berkeley National Laboratory Animal Welfare and Research Committee. Tissue collection for all developmental stages was performed using C57BL/6N strain *Mus musculus* animals. For E14.5 and P0, breeding animals were purchased from both Charles River Laboratories (C57BL/6NCrl strain) and Taconic Biosciences (C57BL/6NTac strain). For all remaining developmental stages, breeding animals were purchased exclusively from Charles River Laboratories (C57BL/6NCrl strain). Wild-type male and female mice were mated using a standard timed breeding strategy. Embryos and P0 pups were collected for dissection using approved institutional protocols. Embryos were excluded if they were not at the expected developmental stage. To avoid sample degradation, only one embryonic litter or P0 pup was processed at a time, and tissue was kept ice-cold during dissection. Collection tubes for each tissue type were placed in a dry ice ethanol bath so that tissue samples could be flash-frozen immediately upon dissection. Tissue from multiple embryos was pooled together in the same collection tube, and at least two separate collection tubes were collected for each tissue-stage for biological replication. Experimenter blinding was not performed for tissue dissection, as there were no separate treatment and control groups being assessed. Randomization was not feasible given the scale of production. Tissue was stored in a freezer at −80 °C or on dry ice until further processing. A step-by-step protocol for tissue collection, including detailedinformation about how embryonic stage was determined, can be found on the ENCODE Project website at https://www.encodeproject.org/documents/631aa21c-8e48-467e-8cac-d40c875b3913/@@download/attachment/StandardTissueExcisionProtocol_02132017.pdf.

### ChIP–seq data generation

The complete ChIP–seq data series includes more than 66 billion sequencing reads from 564 ChIP–seq experiments, each consisting of two biological replicates derived from different embryo pools (*n* = 1,128 replicates total). ChIP–seq experiments for all marks and tissues from E11.5 to P0 were performed as previously described^5^. The ChIP–seq protocol was modified slightly for all E10.5 experiments owing to the low amount of input (micro-ChIP–seq). Detailed protocols for both standard and micro-ChIP–seq, including antibodies used and antibody validations performed, are available at https://www.encodeproject.org/ associated with each experiment described here. They can also be found at the links below.

#### Standard ChIP–seq (E11.5–P0)

Tissue fixation & sonication: https://www.encodeproject.org/documents/3125496b-c833-4414-bf5f-84dd633eb30d/@@download/attachment/Ren_Tissue_Fixation_and_Sonication_v060614.pdf. Immunoprecipitation: https://www.encodeproject.org/documents/89795b31-e65a-42ca-9d7b-d75196f6f4b3/@@download/attachment/Ren%20Lab%20ENCODE%20Chromatin%20Immunoprecipitation%20Protocol_V2.pdf. Library preparation: https://www.encodeproject.org/documents/4f73fbc3-956e-47ae-aa2d-41a7df552c81/@@download/attachment/Ren_ChIP_Library_Preparation_v060614.pdf.

#### Micro-ChIP–seq (E10.5)

Tissue fixation & sonication: https://www.encodeproject.org/documents/1fcaab50-6ca0-4778-88cb-5f6b85170d21/@@download/attachment/Ren%20Lab%20ENCODE%20Tissue%20Fixation%20and%20Sonication%20Protocol%20MicroChIP.pdf. Immunoprecipitation and library preparation: https://www.encodeproject.org/documents/18580e80-0907-4258-a412-46bcc37bd040/@@download/attachment/Ren%20Lab%20ENCODE%20Chromatin%20Immunoprecipitation%20Protocol%20MicroChIP.pdf.

### ATAC–seq data generation

The full ATAC–seq data series includes more than 7 billion sequencing reads from 66 experiments (*n* = 132 replicates total). Our ATAC–seq procedure is based on a previously published method^8^, with modifications to optimize for frozen tissue. In brief, tissues were pulverized with mortar and pestle in liquid nitrogen, and then nuclei permeabilization was performed by resuspension in a nuclei permeabilization buffer (PBS, 1 mM DTTm 0.2% IGEPAL-CA630, 5% BSA, 1× cOmplete protease inhibitor cocktail), and incubation with very gentle rotation at 4 °C. Our full ATAC–seq protocol is available via the ENCODE data portal here: https://www.encodeproject.org/documents/4a2fc974-f021-4f85-ba7a-bd401fe682d1/@@download/attachment/RenLab_ATACseq_protocol_20170130.pdf. We required a minimum of 20 million usable ATAC–seq read pairs per data set and a minimum fraction of read overlapping TSS (FROT) of 0.1 (Extended Data Fig. 3). We use FROT as a measure of signal-to-noise ratio in ATAC–seq data sets because TSSs are widely marked by open chromatin, even in tissues in which the gene is not expressed. We calculate FROT for each library as the number of reads that map within 1 kb of a GENCODE v4 TSS, divided by the total number of usable reads. ATAC–seq data are highly reproducible between biological replicates of the same tissue-stage as measured by Pearson and Spearman correlation (Extended Data Fig. 3). In addition, multidimensional scaling analysis of ATAC–seq enrichment across identified peaks confirms that the samples tend to cluster primarily by tissue types and then by developmental stage (Extended Data Fig. 3).

### ChIP–seq data processing and analysis

#### Uniform processing pipeline

Histone ChIP–seq data were analysed using a software pipeline implemented by the ENCODE Data Coordinating Center (DCC) for the ENCODE Consortium. Each step of the pipeline corresponds to a script written in the Python programming language that assembles the input files, runs external programs (such as the MACS2 peak caller), and calculates quality-control metrics. The methodology is similar to that previously described for ENCODE^62^ with the following modifications: the mapping step used bwa version 0.7.10 and samtools version 1.0, and MACS2 version 2.1.0 was used for signal track generation and peak calling. To ensure adequate sampling of noise for subsequent replicate comparisons, peaks were initially called at a relaxed *P* threshold of 1 × 10^−2^. Such relaxed peak sets were generated for each biological replicate, for the replicates pooled, and for pooled pseudoreplicates of each true replicate (each pseudoreplicate consists of half the reads sampled without replacement). Peaks from the pooled replicate set were retained in the replicated peak set if they overlapped by at least half their length (in bases) peaks from both biological replicates. Additionally, peaks that overlapped both pooled pseudoreplicates were added to the replicated peak set. In this way very strong biological replicates could ‘rescue’ peaks that were only marginal in a second replicate. The pipeline is available to be run on the DNAnexus (https://www.dnanexus.com/) web platform, backed by cloud computing from Amazon Web Services (AWS), and is the same pipeline used for the analysis of all ENCODE histone ChIP–seq experiments. The platform provides both an API for programmatic execution of the pipeline and a web-based interface for interactive execution of the same workflows. ENCODE DCC uses this approach to ensure that primary data from different labs within the Consortium are processed uniformly, and thus to minimize factors that could confound subsequent comparisons^63^. The ENCODE DCC analysed the experiments in parallel and accessioned the results to the ENCODE Portal^62^ (https://www.encodeproject.org/).

#### Analysis of data from individual histone marks

To facilitate comparisons across stages, one peak list per mark per tissue was generated by merging replicated peaks across stages within each tissue. Each peak was then scored using ChIP–seq fold enrichment over input in each stage in the corresponding tissue using bigWigAverageOverBed (https://github.com/ENCODE-DCC/kentUtils/blob/master/bin/linux.x86_64/bigWigAverageOverBed), and using bigwigs from either replicate 1 or replicate 2 as indicated. These values were quantile normalized across stages to eliminate potential confounding effects of biases in the distribution of signal between stages. These normalized score tables (one per mark, tissue, replicate) were used for the analyses below.

#### Comparisons between samples

For correlation between replicates of each experiment, we used Pearson’s correlation as plotted in Extended Data Fig. 2d. Narrow marks (H3K4me3, H3K4me2, H3K27ac and H3K9ac) have tighter peaks of enrichment and tend to correlate more strongly than broad marks (H3K27me3, H3K4me1, H3K9me3 and H3K36me3). For correlation between stages, we used Pearson’s correlation to compare replicates as above for each mark, but comparing all stages to each other within one tissue and one mark. We then categorized these correlations according to how many stages separate the data sets being compared: for example, zero for true biological replicates from the same stage, or seven for comparisons of E11.5 data sets to P0 data sets. These correlations are plotted in Fig. 1e. To further facilitate comparisons across tissues, a similar approach was taken to that described in ‘Analysis of data from individual histone marks’ above, but in this case generating one master peak list per mark by merging replicated peaks across all tissue-stages. As above, each peak was then scored using ChIP–seq fold enrichment over input in each tissue-stage, but in this case using data pooled from both replicates (pooled data). These values were then quantile normalized across tissue-stages, and the resulting master score tables (one per mark) were used for hierarchical clustering performed in R with default parameters. The resulting dendrograms are plotted in Extended Data Fig. 4a. For H3K27ac *k*-means clustering in Fig. 1, one additional data processing step was performed before clustering: across each row, the values were converted to a unit vector in R (*x*/√(sum(*x*^2^)), to prevent overall enrichment level from dominating the clusters. These unit vector values were used only for clustering; the values plotted are the normalized H3K27ac enrichment values from the score tables described above. *K*-means clustering was performed in R with *k* = 8 and default parameters. Rows were ordered within each cluster based on mean normalized enrichment.

#### Principal component analysis

The whole genome was split into 1-kb tiling bins. Average fold enrichment signals were calculated for each bin using the bigWigAverageOverBed. Bins that overlapped a merged peak by a minimum of 20% (reciprocal) were denoted as peak-bins. The average fold enrichment signals from each peak-bin were quantile normalized within a given tissue. The signal strength for each peak was calculated as the sum of the signals of all bins that overlapped that peak. Principal component analysis was performed on the peak signals for each histone mark with the R function ‘prcomp’. PC1, PC2 and PC3 values were plotted for each sample.

#### Metagene profiles

To illustrate the characteristic enrichment patterns at active and silent genes in Extended Data Fig. 1b, we used conservative definitions of ‘active’ genes as reads per kilobase of transcript per million mapped reads (RPKM) > 10 in every tissue-stage evaluated here, and ‘silent’ genes were defined as RPKM < 2 in all tissue-stages. Metagene profiles were plotted with deeptools plotProfile^64^, using data from E15.5 heart.

### ATAC–seq data processing and analysis

#### Uniform processing pipeline

ATAC–seq data were analysed using a standardized software pipeline developed by the ENCODE DCC for the ENCODE Consortium to perform quality-control analysis and read alignment. ATAC–seq reads were trimmed with a custom adaptor script and mapped to mm10 using bowtie version 2.2.6 and samtools version 1.2 to eliminate PCR duplicates. MACS2 version 2.1.1.20160309 was used for generating signal tracks and peak calling with the following parameters: —nomodel —shift 37 —ext 73 —pval 1e-2 -B —SPMR —call-summits. To produce a set of ‘replicated’ ATAC–seq peaks for analysis, the peak calling steps above were performed for each pair of replicates independently as well as for a pooled set of data from both replicates. The intersectBed tool from the bedtools v2.27.1 suite was used to identify a set of replicated peaks, which we define as the subset of peaks called in the pooled set that were also present independently in both replicate peak call sets.

#### d-TAC catalogue

To obtain a uniform d-TAC catalogue that can enable multi-dimensional analysis across all 66 tissue-stages, the aforementioned replicated peak sets for each sample were concatenated, merged, sorted, and then labelled using the mergeBed and sortBed tools from the bedtools v2.27.1 suite. The intersectBed tool was used associate each d-TAC with the original tissue-stages where its constituent peaks were accessible. The catalogue was further categorized as being TSS distal or proximal based on a ±1-kb window around GENCODE v4 TSSs.

To evaluate the sensitivity of our peak calls in detecting potential *cis*-regulatory elements, we calculated the true positive rate, or fraction of peaks recovered, for every applicable tissue-stage with respect to two reference sets: actively transcribed promoters; and enhancers from the VISTA enhancer database (accessed 22 July 2017) with activity at E11.5. Using matched RNA-seq downloaded from https://www.encodeproject.org/, transcripts with counts of ≥10 TPM were classified as actively transcribed for each tissue-stage.

Catalogue specificity was assessed by calculating the true negative rate of each tissue-stage’s d-TACs against GENCODE v4 TSSs that were not accessible to matched DNase-seq from https://www.encodeproject.org/. To further probe the tissue-specificity of the d-TAC catalogue, the overlap between d-TACs for each tissue at E11.5 and enhancers that showed activity in the matching tissue pattern was calculated and compared to a background hit rate of enhancers with activity in any pattern. Enrichment significance was computed using a binomial test.

To calculate enrichment in ChromHMM states, the d-TAC catalogue was overlapped with autosomal ChromHMM state calls for each tissue-stage (pooled or replicate call set, as indicated). Enrichment for a given state *s* in a particular tissue-stage was calculated as the observed number of base pairs of the d-TAC catalogue that overlapped state *s*, divided by the total number of base pairs expected to overlap state *s* on the basis of its genome coverage (total bp coverage of d-TAC catalogue × fraction of genome covered by state *s*).

#### Dynamic d-TACs

To identify differentially accessible d-TACs, for each d-TAC in the uniform catalogue, we counted the number of ATAC–seq reads that overlapped the d-TAC for each tissue-stage and replicate using the coverage function in bedtools v2.27.1. For each tissue, d-TACs at any stage were classified as temporally dynamic if they showed a significant change in accessibility (fold change ≥2, *P* ≤ 0.05) between any sequential stages of development, using DEseq2.

To investigate the relationship between changes in accessibility and changes in chromatin state, the dynamic d-TACs were classified as either gaining (positive log[fold change]) or losing (negative log[fold change]) accessibility. For each tissue-stage-transition (*n* to *n* + 1), these sets of gain- or loss-of-accessibility d-TACs were overlapped with ChromHMM state calls for stages *n* and *n* + 1. Enrichment was calculated by taking the observed fraction of dynamic base pairs that overlapped each combination of states (state at *n*, state at *n* + 1) and dividing by the expected fraction of base pairs that overlapped each state combination based on the dynamic and non-dynamic d-TACs.

To investigate the temporal relationship between H3K27ac and chromatin accessibility, dynamic strong-enhancers (replicated, ChromHMM state U5) at each stage-transition were overlapped against d-TACs for the respective tissue to identify matching enhancers and d-TACs. In cases where more than one d-TAC overlapped an enhancer, the d-TAC with the largest number of overlapping base pairs was selected. The sequential log[fold-change] in ATAC–seq signal was evaluated at every possible stage-transition for these matching d-TACs and a mean was taken. These stage-transitions were converted to ‘offsets’ relative to the strong enhancers and the fold-changes averaged for the purpose of deriving a global trend (that is, for dynamic enhancers at E11.5–E12.5; E11.5–E12.5 is an offset of 0, E12.5–E13.5 is an offset of 1, and so on until E16.5–P0 is an offset of 5). The inverse analysis was also performed to assess the log[fold-change] in H3K27ac at dynamic d-TACs.

#### Correlative d-TAC map

A correlative map between d-TACs was generated for each chromosome by calculating the Pearson correlation coefficient (PCC) for each pair of d-TACs, using the ATAC–seq read counts normalized to RPKM and log_2_-transformed with a small pseudocount. We define ‘correlated d-TACs’ as those in the same TAD (as defined by mouse embryonic stem (ES) cells) with a pairwise PCC ≥ 0.7.

To assess d-TAC correlations as a function of genomic distance, we assigned each d-TAC to a 10-kb bin. For each bin *A*, the correlation was measured between its d-TACs and those of bin *B*, at various distances away ranging from 10 kb to 2 Mb. The average of these correlations across all chromosomes was plotted as a function of distance. Additionally, to investigate the validity of using mouse ES cell TAD boundaries as a constraint for the correlative map, the mean correlations between d-TACs at various genomic distances were compared for pairs located within the same TAD and those not sharing a TAD. The significance of the difference in correlation between intra-TAD and inter-TAD d-TAC pairs was calculated using the Wilcoxon signed-rank test.

#### Enrichment of GWAS catalogue variants in human orthologues of d-TACs

To enable comparison to GWAS of human phenotypes, we used liftOver with default settings to convert d-TACs from mm10 to hg19 genomic coordinates. We then defined novel d-TACs by removing those that overlapped DNaseI hypersensitivity sites from any cell line or tissue in two published data sets^9,36^, one of which included embryonic tissue. We obtained index variants for all traits in the GWAS catalogue (https://www.ebi.ac.uk/gwas/api/search/downloads/full) and retained a unique set of variants that were identified as genome-wide significant (*P* < 5 × 10^−8^) in GWAS of individuals with European ancestry. To obtain a background set of variants for enrichment testing, we used the filtered index variants as the input for SNPsnap^60^, which matches based on (1) minor allele frequency, (2) distance to the nearest annotated gene, (3) gene density in the surrounding region, and (4) number of SNPs in linkage disequilibrium (LD), with the following parameters: European population, ten matched SNPs, exclude HLA SNPs and input SNPs, and report clumping. As GWAS index variants are not necessarily causal and can be in LD with the true causal variant, we next defined loci for all index and matched background variants as all SNPs in high LD (*r*^2^> 0.8) with the variant in European 1000 Genomes^65^ samples using PLINK v1.90p^66^. We then calculated the number of GWAS and background loci with at least one variant that overlapped either all d-TACs or novel d-TACs and used a hypergeometric test to assess the enrichment significance of GWAS loci compared to matched background loci.

#### Enrichment of phenotypes and complex diseases in human orthologues of enhancer d-TACs

To test for enrichment of complex phenotypes and diseases with publicly available summary statistics, we first defined sets of human orthologues of enhancer d-TACs. For each tissue, we collapsed all strong and weak enhancer chromatin states (En-Sd, En-Sp, En-W) across time points and used liftOver to convert genomic coordinates from mm10 to hg19. We then intersected orthologous enhancers with orthologous d-TACs to obtain a set of orthologous enhancer d-TACs for each tissue. We collected summary statistics for 41 human traits and diseases (Supplementary Table 11), converting odds ratios and confidence intervals to log odds ratios and standard errors for binary traits and estimating allele frequencies from the European subset of 1000 Genomes where unavailable from the summary data. We used polyTest^61^ to test for enrichment of variant effects on each phenotype within orthologous enhancer d-TAC annotations with the parameters ‘–univariate–maf 0.05–high-mem’. We used hierarchical clustering on signed −log_10_(*P*) for enrichments that were *z*-score normalized within studies to group similar phenotypes.

#### Cell type enrichment of phenotypes and disease within the mouse forebrain

We obtained the aggregate accessible chromatin peaks for each cell cluster in the P56 mouse forebrain and removed peaks that overlapped promoters (2 kb upstream of mm10 RefSeq TSSs), retaining sets of promoter-distal peaks^38^. For this analysis, we did not restrict peaks to enhancer chromatin states, as doing so would potentially bias results for cell types that were over-represented in the bulk tissue. We converted genomic coordinates for promoter-distal peaks from mm10 to hg19 using liftOver. We then used polyTest to assess cell-type-specific enrichment of phenotypes and diseases that showed at least nominally significant enrichment (*P* < 0.05) in mouse forebrain d-TACs from the previous analysis. We used hierarchical clustering on *z*-score-normalized signed −log_10_(*P*) for enrichment as described in the previous analysis and plotted results for traits that showed at least nominal significance in at least one cell cluster.

### ChromHMM

We note that the chromatin state annotations reported here are specific to this study and are distinct from larger efforts by the ENCODE Data Analysis Center to integrate data from across the entire consortium into a comprehensive ‘encyclopaedia.’ We also note that we excluded E10.5 from the ChromHMM analysis because this stage did not have the full complement of eight histone modification profiles, and testing showed that models with only six marks failed to capture the full set of states derived from eight marks (Extended Data Fig. 7). However, we provide a set of ChromHMM annotations using the six-mark model on E10.5 on our website here: http://renlab.sdsc.edu/renlab_website//download/encode3-mouse-histone-atac/.

#### Generating the model

Chromatin data sets (.bam files) were downloaded from the ENCODE DCC on 15 October 2016. De-duplicated .bam files for each sample, along with their respective input controls, were binarized using the binarizeBam function of ChromHMM, with default parameters. Models considering 2–24 states were learned separately on the two replicates using the LearnModel function, with default parameters. For the rest of the analyses, we leveraged the availability of two distinct replicate time series; namely, we applied the same strategy separately and compared the results a posteriori. The conclusions obtained were invariably consistent, suggesting that the inferences on a single time series (at least in terms of global genomic patterns) are highly reproducible.

#### Identifying the optimal number of chromatin states

We devised two strategies to identify the minimal number of states that captures the combinations of histone modifications present in the data, both of which converged on a 15-state model. First, the ChromHMM CompareModels function was run separately on the two series. This function compares the emission parameters of a selected model to a set of models (in terms of Pearson’s correlation), and outputs the maximum correlation of each state in the selected model with its best matching state in each other model. We used this function to compare the ‘full’ model (the one that considers 24 states) to the states in the simpler models. We then calculated the median correlation of all the 24 states against the simpler models, plotted these numbers against the number of states in the model and looked at the number of states at which both series reached a plateau. As a complementary strategy, the emission probabilities from all the 23 models (considering 2–24 states) from both replicates were clustered together. The rationale behind this strategy is that very similar states across models will tend to cluster together, so there must be an optimal number of clusters corresponding to the optimal number of states in the model. To this end, we applied *k*-means clustering with *k* between 2 and 24, and evaluated the goodness of the separation for each *k* as the ratio between the ‘between sum of squares’ (referred to as Between SS) and the ‘total sum of squares’ (Total SS). Very cohesive, well-separated clusters tend to approach a ratio of 1. Given a value of *k*, the ratio was averaged over one hundred realizations of the clustering. The ratio observed for *k* = 24 was used as a maximum, and the optimal number of states was then defined by the smallest value of *k* that showed a ratio equal to or higher than 95% of the maximum. To compare the eight-mark model to a six-mark model (Extended Data Fig. 7) we used the 66 tissue-stages for which we had the full complement of eight marks, then downsampled to six marks (H3K4me1, H3K4me3, H3K27ac, H3K27me3, H3K9me3 and H3K26me3), and repeated the analyses described above to arrive at an optimal number of states. We then compared the 8-mark 15-state model to 6-mark models with two different numbers of states (11 states and 16 states) representing the minimum and maximum of the optimal range, respectively.

#### Genome segmentation and chromatin state tracking across genomic positions

The segmentation was run separately for each sample, using the MakeSegmentation function of ChromHMM (default parameters) and the model derived from the first replicate. For the final set of replicated state calls we required that a region was assigned to the same state in both biological replicates (within a given tissue and stage). Regions that were not assigned to the same state in both replicates were reclassified as ‘no reproducible signal’ (distinct from state 15: no signal in both replicates). The unionbedg functionality of BEDTools^67^ v2.17.0 was used to keep track of the chromatin state of genomic intervals across a defined set of samples. The total coverage of annotated sequence is 2,725,535,600 bp. This number was used as the denominator to calculate per cent genome coverage from ChromHMM states in the text and figures.

#### Chromatin state trajectories along developmental time

Given a replicate for a defined tissue, all those genomic intervals classified in a specified state (for example, number 5, strong enhancers) at one or more time points were tracked using the approach described above. Considering each pair of adjacent developmental time points, the genomic coverage of each transition between each pair of states was then calculated. The resulting numbers were then normalized on the coverage of the largest transition in the time series under investigation (for example, liver, replicate number 2) and shown as a directed graph.

#### Clustering on enhancer states

After tracking the changes in chromatin state of each genomic base pair in the genome across multiple stages and tissues, the resulting matrix was binarized according to each segment being classified in a specified state (1) or any other state (0). The binary distances between all the pairs of samples considered in each specific analysis were then calculated. These were used either for comparisons or hierarchical clustering (Ward’s method).

#### GO analysis

Functional enrichments through GREAT^68^ were obtained using the greatBatchQuery.py script. The resulting lists were first filtered for the relevant ontologies. After that, only the terms showing a binomial FDR ≤ 0.05 and a regional enrichment equal or higher than twofold were considered. VISTA validated elements were downloaded from https://enhancer.lbl.gov on 17 June 2016. Mm9 and hg19 coordinates were converted to mm10 using liftOver (setting *-*minMatch to 0.95 and 0.1, respectively). VISTA positive elements with any of the following annotations: forebrain, midbrain, hindbrain, neuraltube, limb, facial mesenchyme or heart were considered for the following analysis. Liver was not considered in this enrichment analysis since there are currently fewer than 10 validated elements in VISTA that show reproducible staining in the liver. coverageBed from BEDTools v2.17.0 was used to calculate the coverage of the regions in each state in the E11.5 predictions with each tissue-specific group of VISTA elements. The fraction of bases covered was then normalized to the expected overlap, based on the overall genome-wide coverage of each state. The enrichment for repetitive elements was calculated using the OverlapEnrichment function of ChromHMM.

#### Classification of genes as putative PcG targets

A 2-kb window was defined around the TSS coordinates of all protein coding transcripts in GENCODE^69^ vM9. These 2-kb windows were overlapped with ChromHMM calls (‘pooled’ set) to determine their chromatin state in each tissue and stage. A TSS was classified as active in a given tissue-stage if this 2-kb window overlapped the active promoter state (state no. 1, Fig. 1a), and did not overlap any repressive states (states 3, 13, 14). A TSS was classified as repressed in a given tissue-stage if this 2-kb window overlapped the state characteristic of polycomb-mediated repression state (state 13), and did not overlap any active states (states 1, 2, 4, 5, 6, 7, 10, 12). TSSs that did not meet the criteria for either active or repressed in a given tissue-stage were left unclassified. A gene was classified as a putative PcG target if it had at least one repressed TSS in at least one tissue-stage. To determine whether the genes we identified as putative PcG targets had been identified previously, we compared our data to five published studies examining the genome-wide distribution of H3K27me3 and/or PcG proteins^13,29–32^, including the NIH Epigenome Roadmap data set, which examined at more than 100 human sample types. For refs. ^29,30^, any gene with a TSS annotated as H3K27me3-positive in any sample (irrespective of other marks) was considered a previously identified PcG target. For refs. ^31,32^, any gene classified in one of the ‘PRC’ states in any sample was considered a previously identified PcG target. For ref. ^13^, ChromHMM state calls for 127 human samples were downloaded on 31 March 2018 (‘15_coreMarks_mnemonics’ call set). Putative PcG target genes in this data set were identified as described above for our mouse ChromHMM calls, with the following modifications: the GENCODE v27 annotation set was used for human (gencode.v27lift37.annotation.gtf.gz), and the Polycomb-associated heterochromatin states considered were ‘13_ReprPC’, ‘14_ReprPCWk’, and ‘10_TssBiv’. Any gene with at least one TSS overlapping one of these states in at least one sample was considered a previously identified PcG target. Ensembl v84 was used to match mouse gene IDs with human orthologous gene IDs (attribute = hsapiens_homolog_ensembl_gene). For CpG analyses, a list of CpG Islands with corresponding values (length GC#, CpG#, GC%, CpG%) was downloaded from UCSC Table Browser on 8 January 2018, and overlapped with the list of TSSs using bedtools v2.20.1. If a TSS overlapped more than one CGI, the corresponding values of all overlapping CGIs were combined and associated with the overlapping TSS.

#### Classification of genes as MDG and/or TF

We obtained a list of Mendelian disease genes from https://www.omim.org/^70^ (genemap2.txt, accessed on 14 January 2018). To filter out genes associated with complex diseases or non-disease phenotypes, we performed the following filter steps. 1) We required that the genes be classified as type 3 (the molecular basis of the disorder is known). 2) We required that the gene have at least one associated phenotype that is not in brackets (nondiseases) or braces (multifactorial disorders), or containing a question mark (relationship between the phenotype and gene is provisional). 3) We further required that the human gene Ensembl ID mapped uniquely to one mouse Ensembl ID. 4) Finally, we considered only autosomes, because of the mixed-gender litter pools used for ChIP–seq. These filtering steps led to a set of 3,281 genes that we classified as MDGs. To identify TF genes, we downloaded a list of mouse TFs from the TFClass database^71^ (accessed 18 February 2017). As alternative sources of TF genes to support the TFClass results, we used the DBD: transcription factor prediction database^72^, and genes associated with one or more GO terms containing the phrase ‘TF’ as determined by AmiGO^73^ (accessed on 14 January 2018, taxon_subset_closure_label: *Mus musculus*, document_category: bioentity). AmiGO was also used in this way to identify genes associated with one or more GO terms containing the word ‘development’ (Extended Data Fig. 12). Genes with at least one transcript tagged as a consensus coding sequence (CCDS) in GENCODE were classified as CCDSs in Extended Data Fig. 12.

#### Characterization of dynamic enhancer elements

The temporal dynamic analysis was performed for each tissue separately. First, 1-kb genomic bins that overlapped with regions defined as ChromHMM strong enhancer states in at least one stage were identified. Then we selected dynamic elements (bins) from these strong enhancer bins using the bioconductor LIMMA package^74^ v3.28.21. LIMMA is a package developed for calling differentially expressed genes for microarray but was also adapted for sequencing data with the LOOM functionality. LIMMA was used to call differential enrichment between each adjacent stage comparison (for example, E11.5 versus E12.5, E12.5 versus E13.5, and so on). *P* values were calculated with the eBayes function within LIMMA with trend parameter disabled, and were adjusted using the Benjamini–Hochberg method. A bin was called overall dynamic if its adjusted *P* value was less than 0.05 in any adjacent stage comparison; otherwise it was called a non-dynamic bin. Non-dynamic bins were not included in the following analysis to reduce noise. We performed *k*-means clustering on dynamic bins across stages. The rows (bins) were normalized by dividing by a common value so that the squares of the values sum to 1. The optimal *k* was determined using the elbow method to cut off at the *k* value where percentage of ‘withinness’ values transition from increasing quickly to increasing steadily with larger *k*. The resulting heatmaps of the *k*-means clusters are shown in Fig. 4a and Extended Data Fig. 16. For each of the identified clusters, we performed enrichment testing of GO Biological Processes using GREAT. Over-represented motifs for each dynamic cluster were identified as follows: first, all vertebrate motif position weight matrices (PWMs) were downloaded from the JASPAR TF database and used to scan the peak-bins for motif occurrences with FIMO, MEME suite v4.11.2^75^. For each motif, we computed the odds ratio and the significance of enrichment in each cluster, comparing to a non-dynamic bin pool using Fisher’s exact test. The non-dynamic bin pool was sampled with replacement to match the distribution of average signal strength from the dynamic bins. Following that, significant TF PWMs were grouped in subfamilies using the structural information from TFClass^71^ because they share similar if not identical binding motifs. The top significantly over-represented TFs and their associated subfamilies were reported.

#### Identification of super-enhancers

Super-enhancers were identified using rose v0.1^76,77^ with default parameters for each tissue-stage with H3K27ac signals. Super-enhancers were then combined within the same tissue and across all tissues to generate a non-redundant set of super-enhancers (Extended Data Fig. 14c, Supplementary Table 6).

### A TAD-constrained map of enhancer–promoter associations

#### Generating the map

The reproducible strong enhancer calls (state no. 5) were merged using the mergeBed utility from BEDTools v2.17.0. After that, those regions or sub-regions that overlapped the intervals ±2.5 kb from the TSSs of genes in Gencode were excluded from the merged regions using subtractBed from BEDTools v2.17.0. Regions smaller than 2 kb were enlarged to 2 kb from their central coordinate (to allow more robust signal estimation). This resulted in 66,556 putative enhancers. H3K27ac signals at these regions were then quantified using uniquely aligned, de-duplicated reads. These measurements were carried out using the coverageBed utility from BEDTools v2.17.0, then normalized to RPKM according to the sequencing depth of each sample, and log_2_-transformed (zeros were replaced by the smallest detectable value larger than zero). The mRNA expression of protein-coding genes was tracked across the 66 samples. Small and non-coding RNAs were excluded from any subsequent step by considering only those genes with a GENCODE biotype supporting protein-coding functionality. FPKM were log_2_-transformed (zeros were replaced by the smallest detectable value larger than zero). For each TAD defined in the genome of mouse ES cells^45^, the putative enhancers and genes were retrieved. All the enhancer–gene pairs within the TAD were then evaluated in terms of SCC between the H3K27ac pattern of enrichment and the mRNA expression across the samples. Each gene was assigned to the putative enhancer showing the highest value of SCC. To attach *P* values to these correlations, a null distribution was estimated empirically, by calculating the SCC of the enhancer with all the genes on the chromosome. Two strategies were used to estimate a *P* value: 1) a *z*-score was calculated by subtracting the mean and dividing by the standard deviation of the null, and the corresponding *P* value was then calculated using the pnorm function in R; 2) an empirical *P* value was defined as the number of times an equal or better than the observed SCC was found in the null. Only those putative enhancers showing a *P* value ≤ 0.05 (for both strategies) and an SCC ≥ 0.25 were retained. Two maps were independently derived from the two biological replicates. Only these overlapping associations were used for further evaluation and analyses.

#### Validation of the enhancer–gene map using published chromatin conformation data

Capture-C interaction data from the developing limb and brain^48^ were retrieved from the GEO (GSE84792). Chromatin interaction analysis by paired-end tag sequencing (ChIA–PET) interactions at sites bound by the cohesion subunit SMC1A in the developing limb^49^ were retrieved from Supplementary Table 2 of the original publication. Enhancer–gene contacts in fetal liver cells as inferred from Capture HiC^50^ were downloaded from ArrayExpress (E-MTAB-2414). In all cases, mm9 coordinates were mapped to mm10 using liftOver. For each published data set, only those regions in the enhancer–gene map that overlapped any experimentally validated interaction were retained. The fraction of interactions showing experimental support was then calculated for both the gene assigned by correlation and the nearest RefSeq gene.

#### Mapping of mouse enhancer–gene map to human

The putative enhancer regions were mapped to the human genome (hg19) using liftOver, with a strategy similar to previous reports^78^. Each region was required to both uniquely map to hg19, and to uniquely map back to the original region in mm10, with the requirement that ≥50% of the bases in each region were mapped back to mouse after being mapped to human. For each enhancer–gene pair, the orthologous human gene was inferred using BioMart^79^ (Ensembl version 87; from http://www.ensembl.org/biomart/martview, Filters -> Multiple Species Comparisons -> Attributes -> Homologues -> Mouse Orthologues). The orthologous pairs were also required to share the same TAD in human (TADs derived from human ES cells^45^). Three thousand, five hundred and seventy of the genes in our mouse map had a human orthologue (gene) and at least one linked enhancer with an alignable region in the human genome (residing in the same human TAD). Of the 17,689 putative enhancers that were successfully mapped to hg19, 12,564 were assigned to genes with an unambiguous homologue in human.

#### Validation of the enhancer–gene map using published eQTL–gene associations

Single-tissue eQTL–gene associations generated by the GTEx consortium^80^ were downloaded from the GTEx portal (http://gtexportal.org, release v6p). Only those tissues with more than 750,000 annotated eQTLs were considered. A control set of enhancer–gene associations matching the size and the TSS-distance distributions of the real enhancer–gene map was generated. In brief, for each enhancer–gene pair, the distance between the TSS of the gene and the central coordinate of the enhancer was calculated; after that, a region the same size of the enhancer centred at the same distance to the TSS of the gene but on the opposite side of the enhancer was picked as a control set. For the eQTL analysis, the fraction of eQTLs supported by enhancer–gene pairs was then calculated for ten equal-sized bins based on the distance between the enhancer and the TSS of the gene. The same procedure was applied to the nearest gene. The fraction of associations supported by eQTLs was then calculated, separately for the two groups and for each one of the ten bins. These numbers were used to derive a *P* value for each bin using Fisher’s exact test. For this analysis, we considered only those eQTLs derived from human tissues for which the equivalent tissue was profiled in this study (brain, heart, liver, lung, stomach and small intestine).

#### Comparisons to publicly available maps of enhancer–gene associations

Data sets from ref. ^6^, GeneHancer^81^, JEME^82^, and RIPPLE^83^ were downloaded and consistently re-mapped to the hg19 genome using liftOver. Mapping of enhancer–gene associations between different maps was performed using closestBed from BEDTools v2.17.0.

### Transgenic reporter assays

#### Prospective testing of elements

Names for functionally validated enhancers used throughout this work (mm numbers) are the unique identifiers from the VISTA Enhancer Browser (https://enhancer.lbl.gov/)^34^. Enhancers were selected for testing as follows: The H3K27ac peak calls for three tissues (E12.5 heart, forebrain, and limb) were taken from the TSS-distal H3K27ac peaks called using the uniform processing pipeline (mm10-minimal) by the ENCODE DCC (narrow peaks from combined replicates). Peaks for each tissue were ranked by enrichment score (most to least significant). We then selected predicted enhancers from three different bins within each tissue’s ranked list for testing (bins were approximately ranks 1–85, 1,500–1,550, and 3,000–3,050). Loci that were already included in the VISTA Enhancer Browser or that appeared to overlap unannotated promoters were excluded from testing. In total, 150 predicted enhancers were tested, including 60 top ranked candidates (20 per tissue), 45 middle ranked (15 per tissue), and 45 lower ranked candidates (15 per tissue). Transgenic mouse assays were performed in FVB/NCrl strain mice (Charles River) as previously described^52,84^. In brief, predicted enhancers were PCR amplified and cloned into a plasmid upstream of a minimal Hsp68 promoter and a *lacZ* reporter gene. Transgenic embryos were generated by pronuclear injection of the resulting plasmids into fertilized mouse eggs. Embryos were implanted into surrogate mothers, collected at E12.5, and stained for β-galactosidase activity. Elements were scored as positive enhancers if at least three embryos had identical β-galactosidase staining in the same tissue. Elements were scored as negative if no reproducible staining was observed and at least five embryos harbouring a transgene insertion were obtained. Genomic coordinates and results for each element are provided in Supplementary Table 10, through the ENCODE project data portal (https://www.encodeproject.org/), and at the VISTA Enhancer Browser website (https://enhancer.lbl.gov/).

#### Retrospective analyses of VISTA elements

Overall, 422, 299 and 414 elements showing activity in forebrain, limb or heart, respectively, were considered. For each ranked list of H3K27ac regions, overlap with positive (those elements showing activity in the same tissue from which the H3K27ac profile was derived) and negative (in all tissues or positive in other tissues) elements was calculated. A spline was used to fit the overlap (0–1 values) against the rank (smooth.spline R function, degrees of freedom (df) = 2), separately for each of the three tissues. To derive estimates of the background validation rates for each tissue, the VISTA elements missed by the H3K27ac profiles were leveraged. Specifically, the number of VISTA elements validated in the tissue and part of this set was divided by the total number of VISTA elements in this set. Validation rates across ranked forebrain VISTA elements were derived using the spline approach described above. Each element was annotated to the best overlapping feature (in terms of signal, or LOD score of the conserved element), for each one of the following categories: H3K27ac enrichment, p300 binding, DNaseI-hypersensitive sites (DHSs), ATAC and phastCons conservation. When available, biological replicates were used to derive separate ranks, then the sum of ranks across them was used to re-rank the elements. DHSs were downloaded from the ENCODE DCC website (accession: ENCSR014SFF) or GEO (accessions: GSM348064, GSM348066, GSM559652). PhastCons conserved elements were download from the UCSC Genome Browser on 24 January 2018 (phastConsElements60way and phastConsElements60wayPlacental)^85^.

### Mapping to repeat element families

As the ENCODE analysis pipeline was focused primarily on uniquely mapped reads, we used a separate approach to study repetitive regions. More specifically, we used a pipeline with two rounds of mapping steps to re-process all the fastq files. In the first round of mapping, sample reads were aligned to the reference genome mm10 using Bowtie with: bowtie hg19 -p 16 -t -m 1 -S–chunkmbs 512–max multimap.fastq input.fastq output.sam^86^. –max is used to separate reads mapping to multiple locations of the genome from uniquely mapped reads. In the second round of mapping, a customized assemblies file was constructed by concatenating genomic instances of each repetitive element subfamily, their 15-bp flanking genomic sequences and a 200-bp spacer sequence in FASTA format^87^. The annotation file for repetitive elements used in this step was downloaded from Repeatmasker.org. A python script was used with parameters as follows: python RepEnrich.py /data/mm10_repeatmasker.txt /data/sample_A sample_A /data/mm10_setup_folder sampleA_multimap.fastq sampleA_unique.bam–cpus 16^88^. The number of reads that mapped to repetitive element subfamilies, repetitive element families, or repetitive element classes was determined using information from both uniquely mapped reads that overlap with repetitive element and non-uniquely mapped reads. As some of the repetitive element subfamilies are very similar to each other, a fractional counts method was used to classify the reads that map to multiple repetitive element subfamilies. It sums reads that map uniquely to a repetitive element subfamily once and counts reads that map to multiple subfamilies using a fraction 1/*n*_s_, in which *n*_s_ is the number of repetitive element subfamilies with which the read aligns. A table of counts that estimate enrichment signal for the repeats classes across different tissues is built as the final output for plotting the figures.

### Data processing in R

Most of the described data processing steps (plotting, statistical tests, calculating correlations and hierarchical clustering) were performed in the statistical computing environment R v.3.3.1 (https://www.r-project.org/).

### Reporting summary

Further information on research design is available in the Nature Research Reporting Summary linked to this paper.

## Online content

Any methods, additional references, Nature Research reporting summaries, source data, extended data, supplementary information, acknowledgements, peer review information; details of author contributions and competing interests; and statements of data and code availability are available at 10.1038/s41586-020-2093-3.

## Supplementary information

Reporting Summary

Supplementary Table 1 This table contains GENCODE TSS with information about Polycomb repression.

Supplementary Table 2 This table contains GENCODE genes with information about Polycomb repression.

Supplementary Table 3 This file contains d-TAC catalog with correlated d-TACs and corresponding Pearson’s correlations.

Supplementary Table 4 This file contains d-TAC human orthologous regions.

Supplementary Tables 5-11 This file contains the following Supplementary Tables. Supplementary Table 5: Enhancer counts for each tissue-stage. Supplementary Table 6: Super-enhancer regions in each tissue. Supplementary Table 7: Dynamic enhancer motifs and GO enrichments related to Extended Data Figure 16. Supplementary Table 8: Enhancer target gene predictions, mouse. Table 8a contains enhancer target gene predictions for replicate 1, 8b contains enhancer target gene predictions for replicate 2, and 8c contains enhancer target gene predictions consistent in both replicates. See Methods section 7. Supplementary Table 9: Enhancer target gene predictions, human. See Methods section 7. Supplementary Table 10: Results of *in vivo* enhancer reporter assays. Supplementary Table 11: GWAS studies examined in Fig. 3j and k.

Supplementary Table 12 This file contains results of retrospective analysis of VISTA database presented in Fig. 5d and e.

Supplementary Table 13 ENCODE accession identifiers for ATAC-seq and ChIP-seq experiments included in this study.

## Data Availability

All raw and processed data can be accessed via the ENCODE Data Collection and Coordination (DCC) website: https://www.encodedcc.org via the experiment IDs listed in Supplementary Table 13.

## Code availability

The ENCODE histone ChIP–seq pipeline is among the collection of ENCODE Uniform Processing Pipelines that can be found here: https://github.com/ENCODE-DCC/ChIP-seq-pipeline.
