# Author Correction: An atlas of dynamic chromatin landscapes in mouse fetal development

**DOI:** 10.1038/s41586-020-03089-4

**Published:** 2021-01-04

**Authors:** David U. Gorkin, Iros Barozzi, Yuan Zhao, Yanxiao Zhang, Hui Huang, Ah Young Lee, Bin Li, Joshua Chiou, Andre Wildberg, Bo Ding, Bo Zhang, Mengchi Wang, J. Seth Strattan, Jean M. Davidson, Yunjiang Qiu, Veena Afzal, Jennifer A. Akiyama, Ingrid Plajzer-Frick, Catherine S. Novak, Momoe Kato, Tyler H. Garvin, Quan T. Pham, Anne N. Harrington, Brandon J. Mannion, Elizabeth A. Lee, Yoko Fukuda-Yuzawa, Yupeng He, Sebastian Preissl, Sora Chee, Jee Yun Han, Brian A. Williams, Diane Trout, Henry Amrhein, Hongbo Yang, J. Michael Cherry, Wei Wang, Kyle Gaulton, Joseph R. Ecker, Yin Shen, Diane E. Dickel, Axel Visel, Len A. Pennacchio, Bing Ren

**Affiliations:** 1 Ludwig Institute for Cancer Research, La Jolla, CA USA; 2 Center for Epigenomics, University of California, San Diego School of Medicine, La Jolla, CA USA; 3 Environmental Genomics and Systems Biology Division, Lawrence Berkeley National Laboratory, Berkeley, CA USA; 4 Department of Surgery and Cancer, Imperial College London, London, UK; 5 Bioinformatics and Systems Biology Graduate Program, University of California, San Diego, La Jolla, CA USA; 6 Biomedical Sciences Graduate Program, University of California, San Diego School of Medicine, La Jolla, CA USA; 7 Department of Pediatrics, University of California, San Diego School of Medicine, La Jolla, CA USA; 8 Department of Cellular and Molecular Medicine, University of California, San Diego School of Medicine, La Jolla, CA USA; 9 Department of Biochemistry and Molecular Biology, Penn State School of Medicine, Hershey, PA USA; 10 Stanford University School of Medicine, Department of Genetics, Stanford, CA USA; 11 Genomic Analysis Laboratory, Salk Institute for Biological Studies, La Jolla, CA USA; 12 Division of Biology and Biological Engineering, California Institute of Technology, Pasadena, CA USA; 13 Howard Hughes Medical Institute, Salk Institute for Biological Studies, La Jolla, CA USA; 14 Institute for Human Genetics and University of California, San Francisco, San Francisco, CA USA; 15 Department of Neurology, University of California, San Francisco, San Francisco, CA USA; 16 US Department of Energy Joint Genome Institute, Berkeley, CA USA; 17 School of Natural Sciences, University of California, Merced, Merced, CA USA; 18 Comparative Biochemistry Program, University of California, Berkeley, Berkeley, CA USA; 19 Institute of Genomic Medicine, University of California, San Diego School of Medicine, La Jolla, CA USA; 20 Moores Cancer Center, University of California, San Diego School of Medicine, La Jolla, CA USA

**Keywords:** Epigenomics, Differentiation

Correction to: *Nature* 10.1038/s41586-020-2093-3 Published online 29 July 2020

In this Article, there are several errors. In Figure 3i, minus signs were missing from the *P* values; they should read *P* = 2.8 × 10^−100^ and *P* = 3.1 × 10^−11^. In Fig. 5e, the maximum value on the horizontal axis should be 2.1 (not 35). In Extended Data Fig. 1a, the text under the table should cite Extended Data Fig. 10 (not Extended Data Fig. 6). In the legend of Extended Data Fig. 4b, the gene name *Nkx2-Nkx5* should read *Nkx2-5*. At the end of the legend of Extended Data Fig. 10a, the sentence “Data shown here and in **d** are from E15.5.” has been added. In the legend for Extended Data Fig. 17a, Fig. 4c should be cited (not Fig. 4b); in the legend for Extended Data Fig. 17g, Fig. 4d should be cited (not Fig. 4c). In Extended Data Fig. 18c, the maximum value on the horizontal axis of both panels should be 2.1K (not 15K). The original Article has been corrected online.

