# Supplementary material for: An atlas of dynamic chromatin landscapes in mouse fetal development
Source: Nature. 2020 Jul 29;583(7818):744–51. doi: 10.1038/s41586-020-2093-3 (PMC7398618; doi:10.1038/s41586-020-2093-3)
Supplement: Supplementary file 1 — Reporting Summary [file 41586_2020_2093_MOESM1_ESM.pdf]

nature research

Corresponding author(s): Bing Ren

Last updated by author(s): Mar 6, 2019

Reporting Summary

Nature Research wishes to improve the reproducibility of the work that we publish. This form provides structure for consistency and transparency in reporting. For further information on Nature Research policies, see Authors & Referees and the Editorial Policy Checklist.

Statistics

For all statistical analyses, confirm that the following items are present in the figure legend, table legend, main text, or Methods section.

n/a	Confirmed	
☐	☒	The exact sample size (*n*) for each experimental group/condition, given as a discrete number and unit of measurement
☐	☒	A statement on whether measurements were taken from distinct samples or whether the same sample was measured repeatedly
☐	☒	The statistical test(s) used AND whether they are one- or two-sided<br>*Only common tests should be described solely by name; describe more complex techniques in the Methods section.*
☐	☒	A description of all covariates tested
☐	☒	A description of any assumptions or corrections, such as tests of normality and adjustment for multiple comparisons
☐	☒	A full description of the statistical parameters including central tendency (e.g. means) or other basic estimates (e.g. regression coefficient) AND variation (e.g. standard deviation) or associated estimates of uncertainty (e.g. confidence intervals)
☐	☒	For null hypothesis testing, the test statistic (e.g. *F*, *t*, *r*) with confidence intervals, effect sizes, degrees of freedom and *P* value noted<br>*Give P values as exact values whenever suitable.*
☒	☐	For Bayesian analysis, information on the choice of priors and Markov chain Monte Carlo settings
☐	☒	For hierarchical and complex designs, identification of the appropriate level for tests and full reporting of outcomes
☐	☒	Estimates of effect sizes (e.g. Cohen's *d*, Pearson's *r*), indicating how they were calculated

Our web collection on statistics for biologists contains articles on many of the points above.

Software and code

Policy information about availability of computer code

Data collection	No software used.
Data analysis	The ENCODE histone ChIP-seq pipeline is among the collection of ENCODE Uniform Processing Pipelines that can be found here: https://platform.dnanexus.com/projects/featured. The code is open-source, and available here: https://github.com/ENCODE-DCC/chip-seq-pipeline. The ATAC-seq data were analyzed using a standardized software pipeline implemented by the ENCODE Data Coordinating Center (DCC) for the ENCODE Consortium to perform quality-control analysis and read alignment. Details in methods, along with versions of specific software packages that were used. The following open source software packages were used in data analysis, as described in methods section: bowtie v2.2.6; samtools v1.2 or v1.0 as indicated in methods; MACS2 v.1.0 or v2.1.1.20160309 as indicated in methods; bedtools v2.17.0, v2.20.1, or v2.27.1 as indicated in methods; R v3.3.1; PLINK v1.90p; SNPsnap (No version available, March 2015 update); polyTest (no information available); bwa v0.7.10; bigWigAverageOverBed (no version available); deeptools v2.5.7; liftOver (no version available); AmiGo v2; MEME v4.11.2; DEseq2 v1.22.0 Rose v0.1; BioMart (no version available, accessed 02/14/2017); greatBatchQuery.py (no version available); chromHMM v1.12; LIMMA v3.28.21.

For manuscripts utilizing custom algorithms or software that are central to the research but not yet described in published literature, software must be made available to editors/reviewers. We strongly encourage code deposition in a community repository (e.g. GitHub). See the Nature Research guidelines for submitting code & software for further information.

Data

Policy information about availability of data

All manuscripts must include a data availability statement. This statement should provide the following information, where applicable:

- Accession codes, unique identifiers, or web links for publicly available datasets
- A list of figures that have associated raw data
- A description of any restrictions on data availability

Raw and processed ChIP-seq data from our study can be accessed via the ENCODE Data Collection and Coordination (DCC) website: www.encodedcc.org. A full list

of the ChIP-seq experiments included in this manuscript can be found at the link below: https://www.encodeproject.org/search/?type=Experiment&assay_title=ChIP-seq&award.rfa=ENCODE3&lab.title=Bing+Ren%2C+UCSD&limit=all. A fill list of ATAC-seq experiments included in this manuscript can be found at the link below:
https://www.encodeproject.org/search/?type=Experiment&award.rfa=ENCODE3&lab.title=Bing+Ren%2C+UCSD&limit=all.&assay_title=ATAC-seq&limit=all.
Additional data files including ChromHMM state calls, dynamic d-TACs, and dynamic enhancers can be found here: http://renlab.sdsc.edu/renlab_website//download/encode3-mouse-histone-atac/.

Field-specific reporting

Please select the one below that is the best fit for your research. If you are not sure, read the appropriate sections before making your selection.

☒ Life sciences ☐ Behavioural & social sciences ☐ Ecological, evolutionary & environmental sciences

For a reference copy of the document with all sections, see nature.com/documents/nr-reporting-summary-flat.pdf

Life sciences study design

All studies must disclose on these points even when the disclosure is negative.

Sample size	Sample sizes were chosen to provide sufficient material for ChIP-seq of multiple histone modifications.
Data exclusions	No data points are excluded, except in rare cases of failed ChIP-seq libraries that did not meet ENCODE quality criteria (https://www.encodeproject.org/chip-seq/histone/), were re-done, and replaced by new libraries from the same biosample.
Replication	2 biological replicates were performed for each experiment, derived from independent embryo pools. Quantitative analyses of reproducibility can be found in Extended data figure 2 and 3.
Randomization	Not randomized. This was not feasible given the scale of tissue dissections and ChIP-seq data production here.
Blinding	Not blinded. This was not feasible given the scale of tissue dissections and ChIP-seq data production here.

Reporting for specific materials, systems and methods

We require information from authors about some types of materials, experimental systems and methods used in many studies. Here, indicate whether each material, system or method listed is relevant to your study. If you are not sure if a list item applies to your research, read the appropriate section before selecting a response.

Materials & experimental systems

n/a	Involved in the study	
☐	☒	Antibodies
☒	☐	Eukaryotic cell lines
☒	☐	Palaeontology
☐	☒	Animals and other organisms
☒	☐	Human research participants
☒	☐	Clinical data

Methods

n/a	Involved in the study	
☐	☒	ChIP-seq
☒	☐	Flow cytometry
☒	☐	MRI-based neuroimaging

Antibodies

Antibodies used

Standard ChIP-seq:
H3K4me1 Abcam ab8895
H3K4me2 Millipore 05-1338
H3K4me3 Millipore 04-745
H3K27ac Active motif 39133
H3K27me3 Active motif 61017
H3K9ac Active motif 39137
H3K9me3 Abcam ab8898
H3K36me3 Abcam ab9050
MicroChIP-seq
H3K4me1 Abcam ab8895 polyclonal
H3K4me3 Cell Signaling 9727 polyclonal
H3K27ac Abcam Ab4729 polyclonal
H3K27me3 Active motif 61017 monoclonal
H3K9me3 Abcam ab8898 polyclonal
H3K36me3 Abcam ab9050 polyclonal
The specific antibody and lot numbers used for each library can be found in the publicly accessible metadata associated with

	each experiment at the ENCODE data portal, here: https://www.encodeproject.org/search/?type=Experiment&assay_title=ChIP-seq&award.rfa=ENCODE3&lab.title=Bing+Ren%2C+UCSD&limit=all
Validation	Validation procedure described here :https://www.encodeproject.org/documents/4bb40778-387a-47c4-ab24-cebe64ead5ae/@@download/attachment/ENCODE_Approved_Oct_2016_Histone_and_Chromatin_associated_Proteins_Antibody_Characterization_Guidelines.pdf<br><br>All validations available at encodeproject.org: https://www.encodeproject.org/search/?type=AntibodyLot&characterizations.lab.title=Bing+Ren%2C+UCSD

Animals and other organisms

Policy information about studies involving animals; ARRIVE guidelines recommended for reporting animal research

Laboratory animals	Mouse tissue collection was performed using C57BL/6NCrl and C57BL/6NTac strain Mus musculus, and breeder mice were purchased from Charles River and Taconic, respectively. Tissue was collected using mouse neonates or embryos for the following developmental stages: E10.5, E11.5, E12.5, E13.5, E14.5, E15.5, E16.5, P0. Biological sex is not visually obvious for these developmental stages and was not assessed. All biological replicates consisted of tissue from multiple embryos and are, therefore, expected to consist of roughly equal numbers of males and females. The number of embryos pooled for each replicate can be found in the publicly accessible metadata associated with each experiment at the ENCODE data portal, here: https://www.encodeproject.org/search/?type=Experiment&assay_title=ChIP-seq&award.rfa=ENCODE3&lab.title=Bing+Ren%2C+UCSD&limit=allTransgenic mouse assays were performed using FVB strain Mus musculus
Wild animals	Study did not involve wild animals.
Field-collected samples	No field samples were collected.
Ethics oversight	All animal work was reviewed and approved by the Lawrence Berkeley National Laboratory Animal Welfare and Research Committee

Note that full information on the approval of the study protocol must also be provided in the manuscript.

ChIP-seq

Data deposition

☒ Confirm that both raw and final processed data have been deposited in a public database such as GEO.

☒ Confirm that you have deposited or provided access to graph files (e.g. BED files) for the called peaks.

Data access links<br>*May remain private before publication.*	https://www.encodeproject.org/search/?type=Experiment&assay_slims=DNA+binding&assay_title=ChIP-seq&award.rfa=ENCODE3&lab.title=Bing+Ren%2C+UCSD&limit=all
Files in database submission	Thousands of files, not feasible to list here.
Genome browser session<br>(e.g. UCSC)	goo.gl/57GK9P

Methodology

Replicates	All ChIP-seq and ATAC-seq experiments were performed on two biological replicates of tissue. For each tissue-stage, we harvested tissues from multiple litters of embryos. Tissue was pooled such that each tissue-stage had two biological replicates derived from different embryos. Each replicate contains tissue pooled from several embryos (precise numbers are provided at encodedcc.org), but the embryos in each replicate are unique to that replicate.
Sequencing depth	A detailed list of ENCODE3 ChIP-seq read depth and other standards can be found here: https://www.encodeproject.org/chip-seq/histone/.
Antibodies	Standard ChIP-seq:<br>H3K4me1 Abcam ab8895<br>H3K4me2 Millipore 05-1338<br>H3K4me3 Millipore 04-745<br>H3K27ac Active motif 39133<br>H3K27me3 Active motif 61017<br>H3K9ac Active motif 39137<br>H3K9me3 Abcam ab8898<br>H3K36me3 Abcam ab9050<br>MicroChIP-seq<br>H3K4me1 Abcam ab8895 polyclonal<br>H3K4me3 Cell Signaling 9727 polyclonal<br>H3K27ac Abcam Ab4729 polyclonal<br>H3K27me3 Active motif 61017 monoclonal<br>H3K9me3 Abcam ab8898 polyclonal

H3K36me3 Abcam ab9050 polyclonal
The specific antibody and lot numbers used for each library can be found in the publicly accessible metadata associated with each experiment at the ENCODE data portal, here: https://www.encodeproject.org/search/?type=Experiment&assay_title=ChIP-seq&award.rfa=ENCODE3&lab.title=Bing+Ren%2C+UCSD&limit=all

Peak calling parameters

The ENCODE histone ChIP-seq pipeline is among the collection of ENCODE Uniform Processing Pipelines that can be found here: https://platform.dnanexus.com/projects/featured. The code is open-source, and available here: https://github.com/ENCODE-DCC/chip-seq-pipeline. ATAC-seq pipeline: Uniform processing pipeline. ATAC-seq data were analyzed using a standardized software pipeline implemented by the ENCODE Data Coordinating Center (DCC) for the ENCODE Consortium to perform quality-control analysis and read alignment. ATAC-seq reads were trimmed with a custom adapter script and mapped to mm10 using bowtie version 2.2.6 and samtools version 1.2 to eliminate PCR duplicates and mitochondrial reads. To center peaks on the Tn5 cut site, the paired-end read ends were converted to single-ended read ends and the read end was shifted 4bp towards the center of the fragment to account for the Tn5 insertion position by moving the read end towards the center of the fragment. MACS2 version 2.1.1.20160309 was used for generating signal tracks and peak calling with the following parameters: —nomodel —shift 37 —ext 73 —pval 1e-2 -B —SPMR —call-summits. To produce a set of "replicated" ATAC-seq peaks for analysis, the peak calling steps above were performed for each experiment on each pair of replicates independently as well as a pooled set of the two replicates. The intersectBed tool from the bedtools v2.27.1 suite was used to identify a set of replicated peaks which we define as the subset of peaks called in the pooled set, were also present in both of the replicate peak call sets. Any additional code or scripts are available from authors upon request.

Data quality

A detailed list of ENCODE3 ChIP-seq read depth and other standards can be found here: https://www.encodeproject.org/chip-seq/histone/.

Software

The ENCODE histone ChIP-seq pipeline is among the collection of ENCODE Uniform Processing Pipelines that can be found here: https://platform.dnanexus.com/projects/featured. The code is open-source, and available here: https://github.com/ENCODE-DCC/chip-seq-pipeline. ATAC-seq pipeline: Uniform processing pipeline. ATAC-seq data were analyzed using a standardized software pipeline implemented by the ENCODE Data Coordinating Center (DCC) for the ENCODE Consortium to perform quality-control analysis and read alignment. ATAC-seq reads were trimmed with a custom adapter script and mapped to mm10 using bowtie version 2.2.6 and samtools version 1.2 to eliminate PCR duplicates and mitochondrial reads. To center peaks on the Tn5 cut site, the paired-end read ends were converted to single-ended read ends and the read end was shifted 4bp towards the center of the fragment to account for the Tn5 insertion position by moving the read end towards the center of the fragment. MACS2 version 2.1.1.20160309 was used for generating signal tracks and peak calling with the following parameters: —nomodel —shift 37 —ext 73 —pval 1e-2 -B —SPMR —call-summits. To produce a set of "replicated" ATAC-seq peaks for analysis, the peak calling steps above were performed for each experiment on each pair of replicates independently as well as a pooled set of the two replicates. The intersectBed tool from the bedtools v2.27.1 suite was used to identify a set of replicated peaks which we define as the subset of peaks called in the pooled set, were also present in both of the replicate peak call sets. Any additional code or scripts are available from authors upon request.
